# Strategic Prioritization of Mining Policies in Colombia Through The IGOR Hybrid Framework

**DOI:** 10.1007/s00267-026-02500-6

**Published:** 2026-06-11

**Authors:** Patricia Jaramillo-Álvarez, Ricardo Smith, Linda Berrio-Giraldo, Olga Tatiana Araque-Mendoza, Carlos Adrian Correa-Florez, Santiago Arango-Aramburo

**Affiliations:** 1 Universidad Nacional de Colombia–Sede Medellín - Facultad de Minas - Decision Sciences Group, Cra 80 No 65-223, Bloque M8, Colombia; 2 Unidad de Planeación Minero-Energética, subdirección de Minería, Avda. Calle 26 # 69 D-91, Piso 9, Bogotá, Colombia; 3 Universidad Distrital Francisco José de Caldas, Carrera 8 # 40 – 62, Bogotá, Colombia

**Keywords:** Decision support systems, Mining development strategies, Stakeholder analysis, IGOR methodology, Multicriteria decision analysis, Policy prioritization

## Abstract

Strategic decision-making in the mining sector requires navigating complex trade-offs across social, environmental, economic, and governance dimensions—often under deep uncertainty. Traditional prioritization methods, while valuable, may fall short in addressing such multifaceted challenges. This paper introduces IGOR (Importance–Governance–Robustness), an integrated methodology designed to support the prioritization of public policy actions in uncertain and data-scarce contexts. IGOR combines four complementary tools: the Importance-Governance Matrix (IGM), Analytic Hierarchy Process (AHP), Tornado analysis, and Monte Carlo simulation. Together, they provide a framework that balances strategic relevance, institutional feasibility, and resilience to uncertainty. The methodology was applied to Colombia’s National Mining Development Plan 2024–2025 (NMDP), evaluating over 100 actions across strategic objectives and lines. Criteria were developed collaboratively with national stakeholders to ensure contextual relevance and technical soundness. Results demonstrate IGOR’s ability to generate transparent prioritization scores, enabling the identification of high-impact, feasible, and resilient actions. Sensitivity analysis validated the robustness of outcomes under varying stakeholder preferences, reinforcing confidence in the results. IGOR emerges as a decision support tool that is rigorous, adaptable, and replicable, offering a pathway for improving policy planning in the mining sector and other fields facing similar complexity and uncertainty.

## Highlights

- IGOR integrates AHP, IGO, Tornado, and Monte Carlo into one hybrid framework
- Supports robust prioritization of mining policy actions under uncertainty
- Applied to Colombia’s Mining Plan to identify feasible, resilient, high-impact actions
- Results remain stable across varying stakeholder preferences and assumptions

## Introduction

The mining sector plays a critical role in many countries’ economic and social development, contributing significantly to GDP, employment generation, and technological advancement (Soza-Amigo et al., 2021; World Bank, 2013). Considering its importance, difficult interaction with local communities, and environmental impacts, strategic planning in the mining sector is becoming increasingly complex. Governments must balance the economic importance of mining—with its contributions to GDP, employment, and technological progress—with growing social and environmental demands, institutional constraints, and global market volatility (Carmona-García et al., 2017; Choi et al., 2020).

To address these challenges, various methodologies have been applied for sector planning and policy evaluation. Geographic Information Systems (GIS) support spatial decision-making by modeling ore bodies and environmental conditions (Choi et al., 2020). Multi-Criteria Decision Making (MCDM) techniques, such as Analytical Hierarchy Process (AHP) and Technique for Order of Preference by Similarity to Ideal Solution (TOPSIS), help balance conflicting priorities by integrating quantitative and qualitative criteria (Azimi et al., 2011; Fouladgar et al., 2011; Shen et al., 2015). Other tools, such as the Importance-Governance Matrix (IGM) combines the importance of various factors with the governance aspects (Della Spina et al., 2017). Meanwhile, Tornado analysis and Monte Carlo simulation contribute to risk management by analyzing sensitivity and uncertainty (Hoel et al., 2020; Smith & McCardle, 1998).

Recent advances in mining decision-making have expanded traditional MCDM approaches through hybrid frameworks such as Fuzzy-AHP and Fuzzy-DEMATEL, which aim to better capture uncertainty and subjectivity in expert judgments (Paul & Mahapatra, 2025; Paat et al., 2024; Ilbahar et al., 2022; Bakioglu & Atahan, 2021). These approaches represent an important evolution of multicriteria methods by incorporating more flexible representations of expert preferences and complex decision environments. Their growing application in the mining sector reflects an increasing interest in hybrid methodologies capable of addressing multidimensional sustainability challenges. Other recent approaches have combined structured frameworks such as DPSIR with MCDM techniques to better distinguish between external drivers and policy responses in environmental and resource management contexts (Konstadinos et al., 2025).

Additionally, optimization models have been employed to enhance resource utilization and transition planning between mining phases (Afum & Ben-Awuah, 2021), while data mining and business intelligence tools are increasingly used to improve operational forecasting and decision-making (Pejić Bach et al., 2019). Risk assessment and management approaches, such as the Fermatean fuzzy score function-based SWARA method, evaluate environmental and social risks, aligning decisions with sustainable development goals (Deveci et al., 2023; Kloeckner et al., 2021). In addition to multicriteria approaches, mining planning has also been addressed through system dynamics simulation, particularly in contexts characterized by high uncertainty and limited data availability (Elsawah et al., 2017).

Despite the wide range of methodological alternatives available, these approaches are often applied in isolation and fall short of supporting comprehensive and resilient decision-making. This fragmentation becomes particularly problematic during the early stages of policy planning, when resources are limited, information is incomplete, and decisions may have long-term implications. Therefore, there is a growing need for integrated approaches that enable governments not only to identify high-priority actions, but also to assess their feasibility and resilience under uncertainty—without requiring extensive datasets or intensive computational resources.

This study introduces the IGOR methodology (Importance–Governance–Robustness), a hybrid decision-support framework designed to guide the strategic prioritization of mining policy actions. IGOR integrates four complementary tools into a unified process: the Importance-Governance Matrix (IGM), to evaluate relevance and feasibility; Analytic Hierarchy Process (AHP), to structure and weigh decision criteria; Tornado analysis, to assess sensitivity to key uncertainties; and Monte Carlo simulation, to evaluate the robustness of prioritization outcomes under varying stakeholder preferences. IGOR is particularly well suited to policy settings like Colombia’s, where public institutions must act under conditions of limited data and evolving political, legal, and social landscapes. Rather than replacing more advanced simulation-based approaches such as Robust Decision Making (RDM), IGOR serves as a preliminary and complementary step: it enables the early identification of impactful, feasible, and robust policy actions, helping reduce the cognitive and computational burden of subsequent planning efforts.

We apply IGOR as an input for the *2024 – 2035 Colombia’s National Mining Development Plan*, evaluating over 100 proposed actions. This exercise, developed jointly with the Unidad de Planeación Minero-Energética (UPME), shows how IGOR facilitates a transparent, replicable, and evidence-informed prioritization process aligned with national development goals. The structure of this manuscript is organized as follows. Section two outlines the limitation of existing prioritization tools and frames the rationale for integration. Section three presents the IGOR methodology. Section four illustrates its application in Colombia. Finally, section five offers concluding insights and future research directions.

## Limitations of Existing Prioritization Tools and the Need FOR Integration

Decision-making processes in complex fields such as urban planning, resource management, and industrial development often involves navigating multiple criteria and conflicting objectives (Della Spina et al., 2017; Döll et al., 2008; Fritzsche et al., 2018). Over the years, several prioritization techniques have been developed to support these processes, each offering specific strengths while presenting critical limitations. For example, the Importance-Governance Matrix (IGM) offers a dual perspective by assessing the significance of policy actions and the institutional capacity to implement them. This approach is valuable for contextualizing decision-making, yet its lack of standardization and the inherent subjectivity in evaluating governance capacity can introduce inconsistencies and bias (Della Spina et al., 2017; Döll et al., 2008; Fritzsche et al., 2018).

Multi-Criteria Decision Making (MCDM) techniques, such as Analytical Hierarchy Process (AHP) (Saaty, 1980) and TOPSIS (Yoon & Hwang, 1995), are commonly used to integrate quantitative and qualitative information. AHP decomposes complex problems into hierarchical structures, facilitating prioritization through pairwise comparisons (Mardani et al., 2015; Taherdoost, 2017) and has been applied in fields such as construction management, urban planning, and business decision-making (Abdrabo et al., 2020; Canco et al., 2021; Erdogan et al., 2017).

Recent applications in the mining sector have extended these approaches by incorporating sustainability dimensions and addressing complex risk environments. Studies highlight the importance of integrating non-economic criteria—such as environmental and social considerations—into decision hierarchies (Berberoglu et al., 2024), while AHP-based frameworks have been applied to high-risk mining contexts to evaluate safety-related indicators combining qualitative and quantitative information (Li et al., 2026). Complementary work has also integrated decision frameworks with dynamic models to enhance predictive capabilities in complex mining systems (Zhang et al., 2025), reinforcing the shift toward hybrid and multi-dimensional approaches. However, AHP’s dependence on subjective judgment and its computational complexity with large numbers of criteria remain significant drawbacks (Aguarón et al., 2019; Pamučar et al., 2018).

Other studies have sought to address some of these limitations through hybrid approaches such as Fuzzy-AHP and Fuzzy-DEMATEL, which aim to better capture uncertainty and subjectivity in expert judgment (Paul & Mahapatra, 2025; Paat et al., 2024; Ilbahar et al., 2022; Chalgri et al., 2025; Balusa et al., 2024). These methods extend traditional MCDM frameworks by incorporating fuzzy logic to model vagueness in preference elicitation and improve consistency in decision-making processes, particularly in contexts involving sustainability assessment and mining method selection under uncertainty. However, these approaches primarily conceptualize uncertainty as epistemic uncertainty—i.e., imprecision in expert knowledge—rather than as exogenous factors that affect system performance. In the context of mining policy, decision-making is also shaped by external drivers such as market volatility, geopolitical dynamics, and regulatory changes, which are not fully addressed by these methods.

TOPSIS, evaluates alternatives based on their distance from ideal and anti-ideal solutions, offering simplicity and ease of implementation (Axelsson et al., 2021; Chen, 2020). Despite its widespread use in areas such as healthcare, urban planning and supplier selection (Axelsson et al., 2021; Chen, 2020; Rana et al., 2023), its assumptions of linearity and sensitivity to the choice of metrics can undermine reliability, particularly in dynamic policy environments (Chen, 2020; Karatas et al., 2018).

Optimization models offer mathematical rigor in identifying the best alternative by maximizing or minimizing objective functions under defined constraints. These models are highly effective in sectors like transportation and infrastructure planning but depend heavily on precise input data and can be computationally intensive (Li Zongzhi & Sinha Kumares C., 2009; Zhang & Alipour, 2020). Likewise, Geographic Information Systems (GIS) enhance spatial analysis by integrating diverse data layers, supporting applications in urban planning, environmental management, and disaster risk reduction (Abdrabo et al., 2020; Hamdan et al., 2025). However, GIS effectiveness relies on accurate input data and requires specialized expertise for interpretation.

To address uncertainty, Tornado analysis and Monte Carlo Simulation are frequently employed. Tornado analysis facilitates visual identification of the most influential variables affecting outcomes, although it remains primarily descriptive and does not account for interactions among variables (Cerreta & De Toro, 2012; Hoel et al., 2020; Yin et al., 2017). Monte Carlo Simulation, by contrast, offers a probabilistic exploration of possible scenarios, enhancing robustness in decision-making under uncertainty. However, its implementation is data-intensive and computationally demanding, and it may obscure causal relationships due to its abstract nature (Duintjer Tebbens & Thompson, 2018; Smith & McCardle, 1998; Zhu et al., 2020).

System dynamics is widely recognized as a powerful tool for analyzing and managing complex systems, including those in the mining sector (Schwarz, 1978). By modeling nonlinear interactions in simulated environments, it supports the exploration of policy impacts under uncertainty (Arango-Aramburo et al., 2017; Forrester, 1994). Applications include evaluating mining policies in low- and middle-income countries and assessing environmental, economic, and social trade-offs in Colombia’s gold sector (Saldarriaga-Isaza, Arango, et al., 2015). Complementary methods such as experimental economics and participatory scenario planning have also enriched the analysis of stakeholder behavior and long-term planning (Arango-Aramburo et al., 2020; Saldarriaga-Isaza, Villegas-Palacio, et al., 2015). Despite its strengths, system dynamics has limitations when used alone for early-stage policy prioritization. It requires formal modeling, clear system boundaries, and expert input, which can be difficult when actions are poorly defined. Moreover, it is not well-suited for comparing discrete alternatives across multiple criteria or capturing preference variability through probabilistic approaches.

Robust Decision Making (RDM), developed by (Lempert et al., 2003, 2006), is a methodological framework designed to address deep uncertainty. Its primary objective is to prioritize policies, actions, or combinations thereof without relying on precise forecasts of the future—a valuable feature in contexts where data are scarce and the planning horizon is long. The RDM approach involves evaluating a wide range of possible futures through extensive simulations, testing numerous decision alternatives. From these analyses, the most robust and adaptive strategies are identified. However, its practical implementation faces significant challenges: it requires many subjective parameters (often based on expert judgment), involves high computational demands due to the volume of simulations, and produces results that may be difficult to interpret when only limited or imprecise information is available. These limitations can hinder its applicability in real-world scenarios.

While each of these techniques contributes to addressing specific challenges in decision-making, their isolated application often proves insufficient in highly complex and uncertain policy environments. In the mining sector—characterized by long-term planning horizons, fluctuating regulatory landscapes, and socio-environmental challenges—fragmented tools can result in biased prioritization or inadequate strategic alignment. To ensure more coherent, transparent, and resilient decisions, it is essential to adopt an integrated approach that leverages the complementary strengths of diverse methods while compensating for their individual limitations. Such integration not only enhances analytical rigor but also enables the explicit consideration of exogenous uncertainty and supports the prioritization of actionable policy interventions.

This study addresses the limitations of fragmented prioritization approaches by introducing the IGOR methodology—a hybrid framework that integrates classical decision-support tools, including the Importance-Governance Matrix (IGM), Analytic Hierarchy Process (AHP), Tornado-based sensitivity analysis, and Monte Carlo simulations for robustness assessment. Rather than relying on standalone methods, IGOR combines their complementary strengths into a cohesive model that supports early-stage prioritization in complex and uncertain policy contexts. In particular, the methodology serves as a preliminary step to Robust Decision Making (RDM), enabling the identification of key uncertainties and relevant decision alternatives without requiring precise quantitative data. This early-stage filtering reduces both the cognitive and computational burden of subsequent scenario-based analyses. By incorporating IGOR into the planning pipeline, decision-makers can benefit from a more structured, transparent, and adaptive process—particularly relevant for large-scale policy frameworks such as national mining development strategies, where balancing economic, environmental, and institutional priorities is essential.

In the next section, we present the design of this methodology and demonstrate its application in the Colombian mining sector, showing how integration enhances decision support under uncertainty while maintaining transparency, adaptability, and alignment with long-term development goals.

## Methodology

The IGOR methodology (Importance–Governance–Robustness) is proposed as a novel and integrative framework designed to strengthen decision-making in complex and uncertain environments, such as those found in mining sector planning. It brings together four well-established analytical techniques—Importance-Governance Matrix (IGM), Analytic Hierarchy Process (AHP), Tornado sensitivity analysis, and Monte Carlo simulation—into a cohesive, hybrid approach. By leveraging the complementary strengths of these tools, IGOR enables multidimensional and uncertainty-resilient prioritization of policy actions, supporting more informed, transparent, and robust strategic planning.

**Fig. 1:**
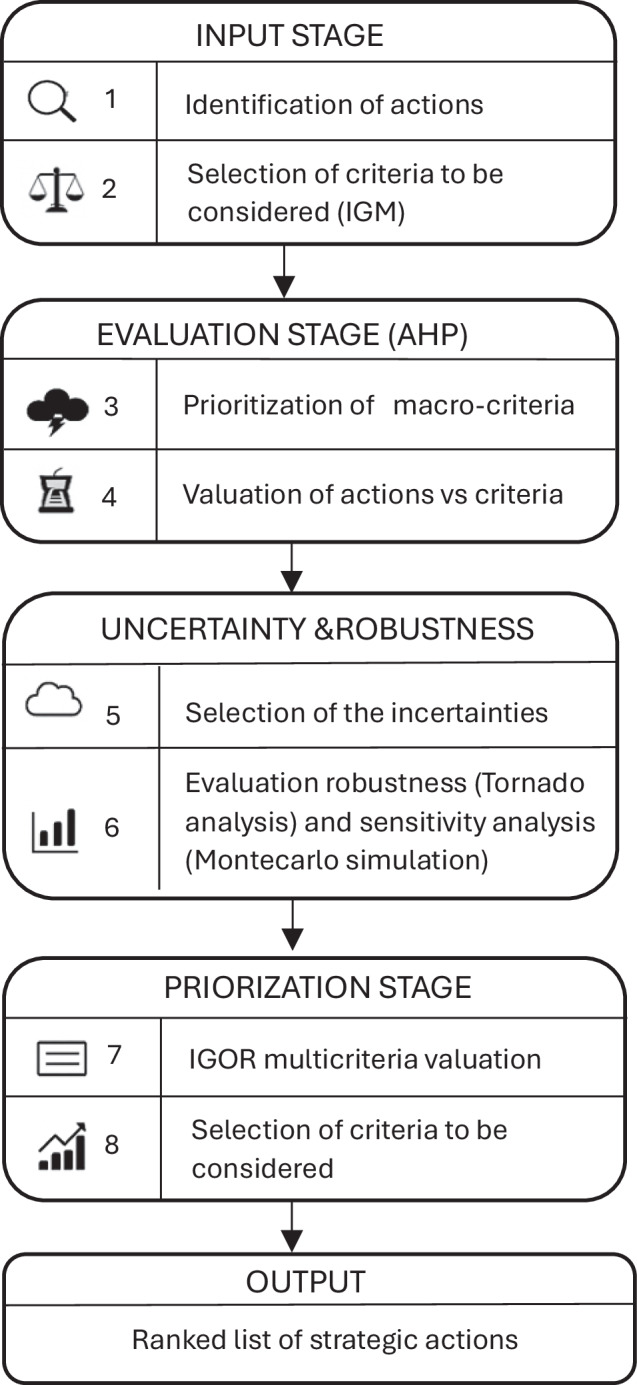
Structured workflow of the IGOR Methodology for policy prioritization

First, *Importance and Governance (IGM)* provide a framework to assess the relevance and feasibility of an action based on its impact on the sector and the institutional capacity to implement it. Second, *Multicriteria Analysis (AHP)* facilitates the hierarchical structuring of evaluation criteria and allows weighting the relative importance of each factor in decision-making. Third, *Robustness Analysis (Tornado)* assesses the sensitivity of actions to key uncertainties, identifying which are more susceptible to contextual changes. Finally, *Sensitivity Analysis (Monte Carlo)* evaluates the stability of action prioritization through the simulation of different scenarios and the variation of criteria weights. The application process of the IGOR methodology consists of eight structured steps, as shown in Fig. 1. This figure illustrates the logical flow of the methodology, from the initial identification of strategic actions and selection of evaluation criteria, through the prioritization and robustness evaluation stages, to the final output: a ranked list of policy actions based on their impact, feasibility, and resilience under uncertainty. Each stage integrates complementary analytical tools (IGM, AHP, Tornado analysis, and Monte Carlo simulation), offering a coherent sequence that guides decision-makers through a transparent and replicable prioritization process. The following sections describe each step in detail.

### Step 1. Identification of Actions to be Prioritized

The first step involves compiling a comprehensive list of potential actions, strategies, or interventions that require evaluation. This process begins with a thorough review of relevant documents, policies, expert opinions, and stakeholder inputs to ensure that all possible actions are considered. The identification phase should be inclusive, capturing a wide range of alternatives without prematurely filtering options based on subjective judgment. To enhance the quality of this process, collaboration with subject matter experts and key stakeholders is essential, as they provide valuable insights into the feasibility, relevance, and potential impact of each action. The output of this step is a structured set of actions that will proceed to the next stages of prioritization, ensuring that the most relevant and impactful options are considered in the decision-making process.

### Step 2. Selection of Criteria to be Considered

**Fig. 2:**
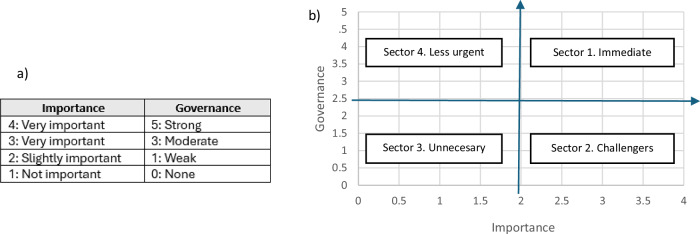
Evaluation scale **a** and cartesian plane **b** of IGO method

Usually, the IGM method requires experts to directly define the importance and governance of an action using the evaluation scale. The importance of an action is assessed based on its relevance and positive impact on the sector, while governance is determined by the capacity to implement and enforce the action effectively. The evaluation scale is presented in Fig. 2-a and the assigned values for each strategy are then plotted on a cartesian plane (Fig. 2-b), allowing a visual representation of their relative importance and governance levels.

The graph is divided into four quadrants that define the prioritization of alternatives:

- Sector 1 – Immediate Strategies: Actions with high governance and high importance. These strategies are well within control, and their execution will have a significant impact. They are considered high-priority.
- Sector 2 – Challenging Strategies: Actions with high importance but low governance. These strategies are critical but difficult to implement due to institutional or operational constraints. The challenge lies in improving governance to facilitate their execution.
- Sector 3 – Unnecessary Strategies: Actions with low governance and low importance. These are the least relevant and have the lowest priority.
- Sector 4 – Less Urgent Strategies: Actions with high governance but low impact. While they are feasible to implement, their effect on the system is not significant.

However, policy evaluation in complex sectors, such as mining, requires a more comprehensive approach that accounts for economic benefits and social and environmental impacts, which are often not explicitly considered in a direct assessment.

To enhance decision-making, we propose a Multicriteria Analysis (MCA) that evaluates each action through a structured hierarchy of criteria, rather than assigning direct scores for importance and governance. This approach enables a more holistic assessment of Importance, Governance, and Robustness. From this point forward, these will be referred to as Level 1 criteria or macrocriteria, further broken down into subcriteria using the Analytic Hierarchy Process (AHP).

The AHP method, developed by Saaty (1980), organizes the decision-making problem into criteria and subcriteria to group similar elements, helping decision-makers clarify and structure the issue more effectively. AHP is one of the most well-known and widely used decision-making methods due to its simplicity, and various software and libraries facilitate its calculations.

The AHP multi-criteria method uses hierarchical trees of criteria to support the analysis. Each of the macro-criteria (Importance – Governance – Robustness) will be assessed by the impact of each action on certain criteria of level two and three. The criteria selection will be the result of discussions by a multidisciplinary team of experts in prioritization and decision-making methodologies and experts in the sector of interest for analysis. It is important that the criteria cover different aspects (i.e. economic, social, environmental) that are assessable, that allow actions to be differentiated, that are relatively pure, and independent.

To compare and prioritize different actions with AHP multi-criteria analysis, the evaluation of the multiple criteria can be both quantitative and qualitative. They can be measured using numerical value intervals, ordinal scales or verbal expressions, depending on the information available.

### Step 3. Prioritization of Importance and Governance Criteria

The goal of this step is to assign a relative level of importance to each criterion based on expert judgment. This is achieved through the allocation of importance levels, which are categorized as follows: (1) Not important, (2) Barely important, (3) Moderately important, (4) Very important, and (5) Absolutely important. To ensure transparency and reproducibility, these linguistic categories are mapped onto a normalized numerical scale ranging from 0 to 1, with five discrete levels corresponding to each category. In this scale, 0 represents “not important” and 1 represents “absolutely important,” with intermediate values capturing gradual levels of relevance.

The importance level assigned to a criterion reflects its relative significance within the group of criteria being evaluated. This means that, while all criteria might be considered very important, the exercise requires differentiation: Which criterion is less important? Which one is more critical than the rest? In some cases, a criterion may even be deemed entirely irrelevant to a specific strategic objective. This process ensures a structured and transparent approach to identifying the relative priorities of each criterion, enabling more effective decision-making aligned with the overarching objectives.

### Step 4. Valuation of Actions vs. Criteria of Importance and Governance

The IGOR evaluation is a multi-criteria analysis designed to assess each action associated with a specific objective through three macro-criteria: Importance, Governance, and Robustness. For each macro-criterion, experts evaluate the extent to which each action contributes to each criterion by assigning a percentage ranging from 0% to 100%. This percentage represents the positive impact the action is expected to have on the criterion. The detailed methodology for assessing Robustness macro-criterion is explained in a later section.

These evaluations are organized into matrices for each macro-criterion, where rows correspond to the actions being assessed, and columns represent the criteria under evaluation. This structured approach facilitates a comprehensive and systematic analysis, ensuring that the contributions of each action to strategic objectives are thoroughly quantified and compared.

### Step 5. Selection of the Most Relevant Uncertainties

The purpose of this step is to identify the uncertainties that could have the greatest impact on the performance of the proposed actions in the mining sector. Given the complexity and dynamic nature of this industry, uncertainties may arise from regulatory changes, economic fluctuations, environmental factors, technological advancements, geopolitical tensions, or social dynamics. These uncertainties introduce risks that could significantly alter the feasibility, effectiveness, and long-term success of mining policies and strategies. Therefore, a structured approach is required to systematically assess their potential impact.

To ensure a robust and comprehensive evaluation, uncertainties are assessed through structured expert elicitation. In the IGOR framework, this stage relies on technically informed actors capable of providing forward-looking judgements in contexts where data are limited and risk assessment is inherently probabilistic.

Experts are asked to evaluate each identified uncertainty based on two key dimensions: its level of impact on the mining sector (considered as a threat) and its likelihood of occurring in the future. The predefined uncertainties are categorized into natural, legal, geopolitical, technical, financial, and social risks, as outlined in Table 1. For example, natural disasters such as landslides, floods, and earthquakes could severely disrupt mining activities, while regulatory instability may create an uncertain environment for investors. Geopolitical conflicts could lead to supply chain disruptions and price volatility, whereas social resistance to mining could increase operational barriers.

**Table 1:** Key uncertainties affecting the Mining Sector and their potential impacts

Category	Uncertainty	Description
Natural	Natural disasters	Severe landslides, floods, or earthquakes affecting the mining sector
Legal	New legal requirements	High instability in regulations for investors.
Geopolitical	Global geopolitical instability	Wars or conflicts cause supply chain disruptions and price fluctuations.
Technical	Availability of discovered minerals	Limited availability of economically, socially, and environmentally exploitable materials.
	International demand	Significant decrease in demand for strategic minerals.
Financial	International prices of critical minerals	High price volatility
Social	Social opposition to mining	Increased social barriers to mining operations
	Armed conflict	Renewed intensification or armed conflict

Each uncertainty is rated according to its impact level, ranging from insignificant (1) to catastrophic (5), and its probability of occurrence, classified into six predefined levels, from rare (<20%) to almost certain (80–99%). This evaluation allows for a prioritization of uncertainties based on their potential disruptive effects, enabling decision-makers to develop proactive mitigation strategies.

Table 2, commonly referred to as the heatmap matrix, presents the priority rules determined by the intersection of impact and probability ($i* {p}$). The priority levels are categorized into four groups: extreme (represented in red), high (represented in yellow), moderate (represented in blue), and low (represented in green). Uncertainty factors classified as extreme priority are selected for robustness analysis, as they pose the most significant risks to the effectiveness of mining policies. A robust alternative is characterized by its lower sensitivity to uncertainty, greater tolerance to unexpected changes, and its ability to perform satisfactorily relative to a predefined threshold or goal across the most possible scenarios of various uncertainty variables.

**Table 2:**
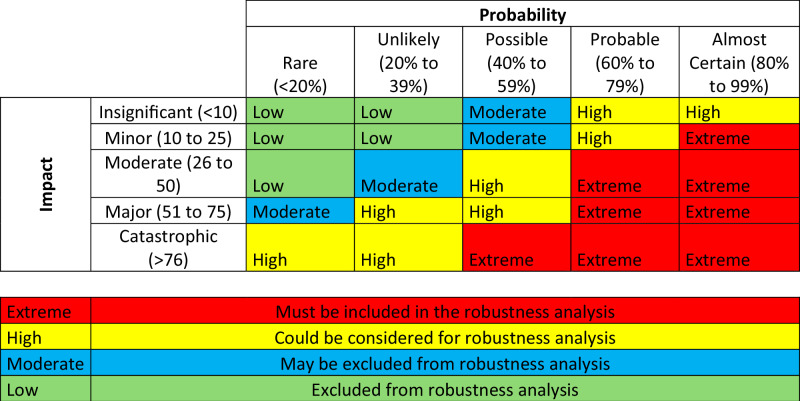
Classification of impact and probability levels and corresponding priority rules. Heatmap Matrix: Priority rules

### Step 6. Evaluation of the Robustness and Sensitivity of Each Action for Relevant Uncertainties

This step evaluates how each action performs under conditions of deep uncertainty, focusing specifically on sensitivity and robustness. The objective is to determine which actions are more vulnerable to uncertainty and which remain consistently effective across different future scenarios. Given that not all uncertainties impact every action equally, the analysis includes only those uncertainties classified as *extreme priority* in the previous step.

For each action $i$, the same technically qualified expert group involved in the IGOR evaluation process assess how different states of a given uncertainty $j$ influence the achievement of the corresponding strategic objective. To structure this evaluation, two boundary scenarios are defined:

- Best-case scenario: The most favorable outcome of uncertainty j,
- Worst-case scenario: The most adverse possible condition of uncertainty j.

For example, regarding *global geopolitical instability*, the worst-case scenario may involve severe international crises affecting major mineral suppliers and consumers, while the best-case scenario reflects international stability and trade cooperation.

Due to the absence of fully developed quantitative models, experts provide qualitative assessments of expected performance using a scale from 0% (no achievement) to 100% (full achievement of the objective). These ratings indicate the expected effectiveness of action i when uncertainty j materializes in either its best or worst form.

Based on these expert evaluations, two key indicators are calculated: sensitivity and robustness. Sensitivity (${S}_{{ij}}$) measures how much the achievement of an action fluctuates between the best and worst uncertainty scenarios. If the impact remains stable, the action is considered less sensitive to that uncertainty. The ${S}_{{ij}}$ is calculated as the difference between the highest and lowest achievement levels (Eq. 1).

1 $${S}_{{ij}}={A}_{{ij}}^{B}-{A}_{{ij}}^{W}$$

where ${A}_{{ij}}^{B}$ and ${A}_{{ij}}^{W}$ represent the levels of achievement under the best and worst conditions, respectively. If uncertainty does not directly influence an action, its sensitivity is 0%.

Robustness (${R}_{{ij}}$), on the other hand, evaluates the likelihood of an action being successful despite uncertainty. It is estimated by comparing the best-case achievement level to a predefined success threshold (*T*), which represents the minimum acceptable level to consider an action effective. The ${R}_{{ij}}$ is calculated as follows:

2 $${R}_{{ij}}={\max (A}_{{ij}}^{B}-T,0)$$

It is important to note that the success threshold (denoted as $T$), set to 40% in this study is defined a priori and involves an element of subjectivity, as it reflects a minimum acceptable level of performance under uncertainty. While a value of 50% may represent a neutral benchmark, in this case study it did not provide sufficient discrimination among alternatives. Therefore, a threshold of 40% was adopted to enhance the differentiation of robustness across actions. Different values of *T*may influence robustness rankings and can reflect both methodological considerations (e.g., discriminatory power) and the decision-makers’ level of stringency or risk preference. A sensitivity analysis with respect to *T*could further assess the stability of results and is identified as a relevant extension for future research.

A higher robustness score implies that the action is more likely to succeed despite uncertainty. To aggregate the results across all relevant uncertainties, the average sensitivity and robustness for each action $i$ are computed as:

3 $${S}_{i}=\frac{1}{m}\mathop{\sum }\limits_{j}{S}_{{ij}}$$

4 $${R}_{i}=\frac{1}{m}\mathop{\sum }\limits_{j}{R}_{{ij}}$$

This analysis is fundamental for identifying actions that are highly vulnerable to uncertainty and determining which require greater control and monitoring to mitigate failure risks. The Robustness Matrix (Table 3) complement the Importance and Governance Matrices, forming a comprehensive framework for action prioritization.

**Table 3:** Example of an evaluation matrix for actions vs. robustness criteria

Action	Sensibility ${(S}_{i})$	Robustness (${R}_{i}$)
Action 1	40	20
Action 2	30	16
Action 3	60	15
Action 4	75	2
Action 5	14	5
Action 6	28	7
Action 7	58	18
Action 8	70	32

By integrating both sensitivity and robustness metrics, this step ensures that high-priority actions are not only important and governable but also resilient to severe uncertainties, thus reinforcing the credibility and durability of long-term policy implementation in the mining sector

### Step 7. IGOR Multi-criteria Valuation of Each Action

This step aims to estimate a prioritization index for each strategic action by integrating three core macrocriteria: Importance (I), Governance (G), and Robustness (RO). The integration is conducted through a multi-criteria valuation framework based on the Analytic Hierarchy Process (AHP), allowing for the systematic aggregation of heterogeneous qualitative and quantitative inputs.

Each action $i$ is evaluated across a set of criteria associated with each macrocriterion. For the Importance dimension, the criteria are derived from economic, social, and environmental priorities, such as fiscal revenue, job creation, community development, ecosystem protection, and climate change mitigation. Similarly, the Governance dimension is evaluated based on criteria such as institutional strength, social feasibility, and implementation viability. Finally, the Robustness component is derived directly from the analysis developed in Step 6, which evaluates how each action performs under adverse conditions of extreme uncertainty.

For each macrocriterion $M$, the performance of action $i$ is calculated using a weighted average of its associated criteria $k$, applying normalized weights ${w}_{k}$ that reflects their level of priority. Equations 5–7 follow the standard weighted aggregation structure commonly used in the Analytic Hierarchy Process (AHP) (Saaty, 1990). The general form of this calculation is as follows:

5 $${I}_{i}=\mathop{\sum }\limits_{k}{w}_{k}{C}_{{ik}}^{{Imp}}$$

6 $${G}_{i}=\mathop{\sum }\limits_{k}{w}_{k}{C}_{{ik}}^{{Gov}}$$

7 $${{RO}}_{i}={C}_{i}^{{Rob}}$$

where ${C}_{{ik}}$ represents the score of action $i$ with respect to criterion $k$, and ${w}_{k}$ is the corresponding local normalized weight. To ensure comparability across actions and macrocriteria, all weights are normalized by the sum of the original priority levels of the criteria. For example, if fiscal revenue is assigned a priority weight of 0.5 and other criteria are weighed at 0.75, the normalized weights are adjusted accordingly so that their sum equals one. This approach guarantees consistency across dimensions and avoids overrepresentation of any single criterion.

While both robustness and sensitivity are calculated in Step 6, only the robustness score is included in this multi-criteria valuation due to its strong correlation with sensitivity. Including both measures would introduce redundancy without improving the discrimination power of the index. Therefore, the robustness score ${{RO}}_{i}$ captures the resilience of each action against critical uncertainties, while the sensitivity analysis remains a supplementary component for further interpretation.

Once the individual scores for Importance, Governance, and Robustness are determined, the overall prioritization value for each action is calculated using the IGOR index. Consistent with the AHP aggregation logic at the highest level of the hierarchy (Saaty, 1990), this index is computed as the weighted sum of the three macrocriteria:

8 $${{IGOR}}_{i}={P}_{{Imp}}{I}_{i}+{P}_{{Gov}}{G}_{i}+{P}_{{Rob}}{{RO}}_{i}$$

In alignment with the classical IGO framework, equal weights are assigned to each macrocriterion (i.e., ${P}_{{Imp}}={P}_{{Gov}}={P}_{{Rob}}=0.33$), reflecting their balanced significance in the prioritization process. The resulting IGOR score expresses the aggregated performance of each action, where higher values indicate higher prioritization. This score enables a consistent comparison across all actions within each strategic line, supporting the selection of actions that are not only impactful and feasible but also resilient under conditions of uncertainty.

This methodological step provides a rigorous and transparent basis for ranking strategic actions. By combining structured expert judgments with robust weighting and aggregation procedures, the IGOR index supports informed policy decisions that are better aligned with national development goals, institutional capacities, and the unpredictable dynamics of the mining sector.

### Step 8. Sensitivity Analysis

Given the early stage of the policy design process and the limited availability of empirical data, several assessments involved in the prioritization of actions rely on expert judgment. Within the IGOR framework, expert judgment refers to evaluations provided by technically qualified group of stakeholders directly involved in policy design, sectoral planning, or analytical assessment. These stakeholders typically include institutional decision-makers, sectoral experts, and academic specialists with demonstrated expertise relevant to the policy domain under analysis.

While broader stakeholder participation may inform earlier stages such as action formulation, this stage of the methodology focuses on analytically consistent evaluations carried out by technically informed actors. Consequently, the weights assigned to the macrocriteria—Importance, Governance, and Robustness—reflect the informed preferences of this expert group under conditions of uncertainty.

To evaluate the robustness of the prioritization outcomes considering possible variations in those preferences, a sensitivity analysis was conducted using Monte Carlo simulation. The analysis involved generating $m$ random sets of weights for the three macrocriteria. These weights were drawn from a uniform distribution between 0 and 1, simulating the effect of alternative expert perspectives or future changes in institutional priorities.

For each set of weights, the IGOR index was recalculated for all actions within a given strategic line, resulting in a re-ranking of those actions based on the modified weight configuration. The result of this process is a distribution of $m$ priority ranks for each action, from which descriptive statistics—such as the mean rank or frequency histograms—can be derived. These indicators help assess how stable an action’s priority is across a wide range of plausible weighting schemes.

Importantly, this analysis does not alter the original prioritization results but provides additional evidence to support the robustness of the hierarchy. It enables decision-makers to identify actions that consistently remain among the top priorities regardless of how weights are adjusted, thus reinforcing confidence in their selection under conditions of uncertainty.

## Case study: Prioritization of Actions in Colombia’s National Mining Development Plan 2024–2035

The IGOR methodology was applied to the prioritization of actions within Colombia’s National Mining Development Plan (NMDP) to establish a structured and robust decision-making framework. This case study demonstrates how the proposed methodology integrates multiple analytical approaches to evaluate the importance, governance, and robustness of various policy actions in the mining sector. By systematically assessing these actions under conditions of uncertainty, IGOR helps identify strategic priorities that align with national development objectives while incorporating economic, social, and environmental considerations.

The application of the IGOR framework to the NMDP involved a core technical group composed of five professionals from the UPME Mining Subdirectorate and four academic researchers. The UPME team included specialists in economics, geological engineering, environmental engineering, and sectoral planning, all directly involved in national mining strategy formulation. The academic team contributed expertise in system dynamics, multi-criteria decision analysis, sustainability assessment, and public policy evaluation. This interdisciplinary composition ensured the integration of institutional sectoral knowledge with methodological rigor in structured prioritization and uncertainty analysis. To facilitate its implementation, excel templates were developed, eliminating the need for commercial software and enabling a more accessible evaluation of potential actions in the mining sector (see supplementary material 1 and 2). The following sections detail the outcomes obtained from each methodological step described in the previous section.

### List of Identified Actions (Step 1)

The process of constructing the list of actions was carried out in three sequential stages. This list, which includes a total of 103 actions to be analyzed for prioritization (Appendix A), was developed as an input by the working team of the Mining Subdirectorate of the Unidad de Planeación Minero Energética (UPME). Importantly, the portafolio of actions did not emerge solely from internal technical deliberations. It was derived from the participatory process conducted during construction of NMDP. This process included: (i) inter-institutional working tables involving the Ministry of Mines and Energy (MME), the National Mining Agency (ANM), the Colombian Geological Survey (SGC), and UPME; (ii) structured “strategic bets” submitted by mining association, community councils, environmental organizations, and mining entrepreneurs; and (iii) strategic field visits to mining companies and regional initiatives. These mechanisms ensured that the action portfolio reflected institutional, territorial, productive, and social perspectives prior to the IGOR priorization stage.

The first stage involved identifying and analyzing key elements and foundational documents that provided the conceptual and technical framework for developing the NMDP. These included territorial diagnostics, scenario planning for the mining sector, national development policies, sustainable development goals, institutional guidelines, stakeholder participation, and strategic planning from relevant entities. Each of these components contributed to defining priorities, strategic approaches, and regulatory considerations essential for the NMDP.

In the second stage, the collected information was collaboratively processed within the UPME team through internal technical sessions using grouping methodologies, leading to the identification of seven key strategic themes fundamental to the mining sector’s development. These themes guided the formulation of the plan’s vision, ensuring alignment among different stakeholders. A parallel effort was made to integrate the National Mining Policy (PMN) with the NMDP, ensuring complementary objectives without redundancy. Finally, in the third stage, a structured framework was collectively established to implement the vision by defining four core objectives, each supported by strategic lines aimed at transforming Colombia’s mining sector by 2035. Table 4 presents the objectives and strategic lines consolidated during this process.

**Table 4:** Objectives and strategic lines for NMDP

Objective	Strategic Line
**Objective 1:** Strengthen mining activity planning with social, cultural, environmental, and territorial approaches to ensure the rational use of mineral resources.	Mining Sector Planning.
	Territorial Planning for Mining Activity.
	Conflict Mitigation and the Incorporation of Human Rights, Ethnic, Gender, and Intersectional Approaches in Mining Activities
**Objective 2:** Strengthen institutional capacity in mining activities in order to improve governance, transparency, process excellence, streamlined procedures, and oversight, while promoting stakeholder engagement and institutional communication.	Institutional Governance of the mining sector.
	Interinstitutional Collaboration and Coordination
	Institutional Engagement and Communication.
**Objective 3:** Promote and support the regularization of mining and the implementation of more efficient extraction, beneficiation, and transformation technologies, in order to strengthen the sector and address key challenges such as safety, environmental impact reduction, ecosystem restoration, and community well-being.	Mining Formalization.
	Mining Safety.
	Quality and Efficiency (Best Practices).
	Promotion and Use of Innovation and Technology.
**Objective 4:** Strengthen productive transformation to consolidate value chains based on the responsible exploitation of strategic minerals and to establish new activities that generate added value.	Value Chains
	Economic conversion and diversification

### Selection of Criteria to be Considered (Step 2)

The criteria used for the evaluation and prioritization of actions in Colombia’s National Mining Development Plan were selected through a collaborative analysis process between an academic research team and the UPME. This process began with a broader initial list of criteria, which was refined through technical discussions to ensure they reflected both the objectives of the plan and the specific characteristics of the national context.

The refinement process was carried out through joint working sessions involving members of the UPME Mining Subdirectorate and the academic team. These sessions were conducted in a deliberative and iterative manner, combining face-to-face meetings and virtual technical workshops. During these interactions, alternative criteria were discussed, redundancies were eliminated, and alignment with policy objectives and institutional feasibility was verified before reaching consensus on the final set of evaluation criteria.

**Fig. 3:**
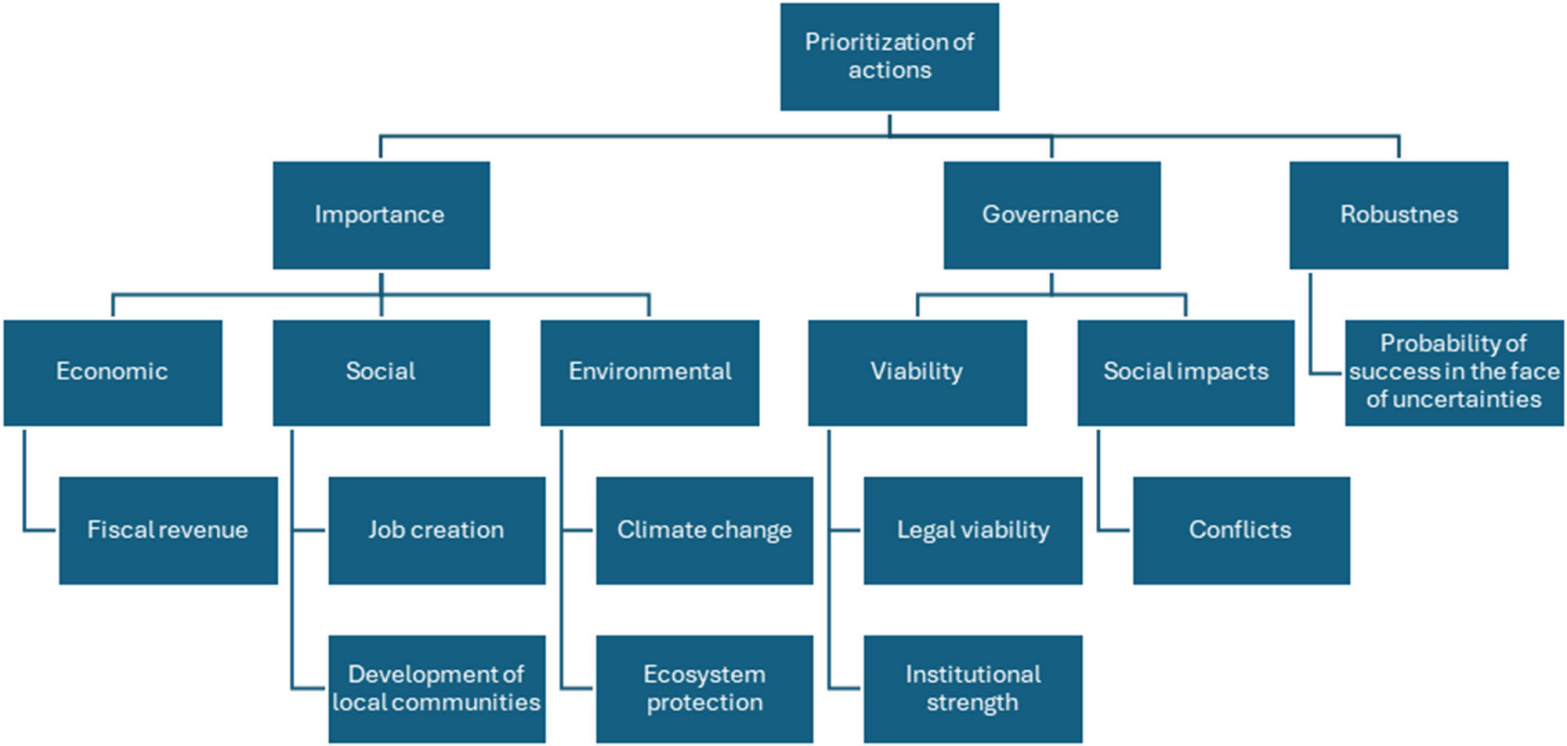
AHP tree for IGOR multicriteria analysis

The selected criteria, presented in Fig. 3, enable each action to be evaluated from an integrated perspective that considers economic, social, environmental, viability, and social impact dimensions, ensuring a holistic and balanced approach. This selection is supported by a growing body of literature applying AHP and related multi-criteria approaches to mining sustainability and impact assessment.

Each action in the plan will be evaluated based on some of the defined criteria, which are structured into two levels. At Level 2, the key dimensions are grouped (economic, social, environmental, viability, and social impact), while Level 3 details specific criteria such as fiscal revenues, job creation, ecosystem protection, legal feasibility, and social conflicts, among others.

The economic dimension considers the contribution of each strategy to fiscal revenues, such as taxes and royalties, which are vital for the financial stability and reinvestment capacity of the state. These criteria are widely recognized as central to national-level mining assessments (Mancini & Sala, 2018; Heydari & Osanloo, 2024).

From an environmental perspective, the criteria prioritize ecosystem protection, evaluating the impact of each strategy on biodiversity, water quality, and air quality in the affected areas, as commonly addressed in sustainability-oriented mining assessments (Heydari & Osanloo, 2025; Paul & Mahapatra, 2025). Another key consideration is the contribution to climate change mitigation, assessing whether the actions reduce greenhouse gas emissions or exacerbate them.

Lastly, the social dimension evaluates the broader societal implications of each action through two complementary subdimensions: “Job creation” and “Development of local communities”. While the former captures the potential to generate employment opportunities, the latter reflects contributions to territorial inclusion, local capacity building, and long-term social development. This distinction is particularly relevant in mining contexts, where specialized skill requirements may limit immediate local hiring. In addition, this dimension considers the potential for social conflict, as well as the capacity of each action to promote inclusion and citizen participation, which are recurrent aspects in the literature on mining-related socio-political dynamics (Paat et al., 2024; Berberoglu et al., 2024).

The Governance macrocriterion evaluates the institutional and regulatory feasibility of implementing each action, recognizing that legal and regulatory viability acts as a critical enabling condition for investment and policy execution at the national level (Paul & Mahapatra, 2025). It is structured around two main components: Viability and Social Impacts. Viability considers the legal and institutional conditions necessary for implementation, including legal feasibility and institutional strength, which determine whether proposed actions can be effectively operationalized regardless of their potential benefits.

Social Impacts assess the potential for conflict generation and the degree to which actions may affect social stability. In this context, the emergence of socio-political conflicts and the absence of a Social License to Operate (SLO) are recognized as major operational and legal bottlenecks that can hinder or even halt mining activities (Mancini & Sala, 2018). Additionally, factors such as corruption, bribery, and the unequal distribution of mining benefits have been identified as key drivers of social tensions and reduced governance capacity (Mancini & Sala, 2018). Together, these subdimensions capture the extent to which proposed actions are implementable within existing regulatory frameworks and institutional capacities, while also considering their potential to generate or mitigate socio-political tensions.

The criteria associated with robustness will be explained in a later section, given its transversal nature in the evaluation. Together, these criteria provide a robust and multidimensional framework for evaluating and prioritizing actions, ensuring alignment with both national development goals and sustainable practices.

### Prioritization of Importance and Governance Criteria (Step 3)

The table of importance levels assigned to the criteria was initially drafted by the academic team as a technical starting point. This preliminary proposal was then jointly reviewed with UPME team through structured working sessions. During these sessions, the proposed importance levels were discussed, validated, and, where necessary, adjusted to reflect institutional priorities and contextual considerations specific to the Colombian mining sector.

In cases where participants found it challenging to assign importance levels directly, the Analytic Hierarchy Process (AHP) methodology was applied to facilitate structured decision-making. Through guided pairwise comparisons exercise, the expert group collectively evaluated the relative importance of each criterion. This process involves constructing an n × n matrix, where *n* represents the number of criteria related to a macro-criterion. Participants assessed the relative importance of each criterion in the rows compared to those in the columns, based on predefined levels. This systematic and quantitative process ensures a more nuanced evaluation, fostering consistency and facilitating consensus among experts.

### Valuation of Actions vs. Criteria of Importance and Governance (Step 4)

The initial evaluations of action performance were prepared by the academic team as a technical baseline and later refined by the UPME team. Rather than unilateral reassessment, the process involved collective deliberation to ensure that the assigned values accurately reflected both methodological consistency and institutional priorities.

During the sessions, the expert group assigned values between 0% and 100% in two matrices (Importance and Governance, respectively), reflecting the positive contribution each action would make to each Level 4 criterion. For example, regarding action “Design and implement a strategy to integrate social and environmental variables into mining planning tools in line with the institution’s mission”—the teams agreed to assign a value of 10% for the criterion “*Fiscal Revenues*,” as this action does not primarily focus on monetary outcomes, and prioritizing environmental protection and social well-being often reduces economic gains. However, for the same action, under the criterion “*Development of Local Communities*,” it was rated 100%, reflecting its significant potential to foster local community benefits. Each action, therefore, has strengths and weaknesses in achieving the specified objective.

This analysis is essential for understanding how each action contributes to the objectives in terms of Importance and Governance. These evaluations serve as key inputs for the final prioritization of actions under each objective and strategic line. Table 5 provides examples of the valuation matrices, where cell intensity in red indicates poorer performance. The Robustness metric is assessed using sensitivity and robustness indicators, and the method for estimating these values will be detailed later, which first requires identifying the most relevant uncertainties.

**Table 5:**
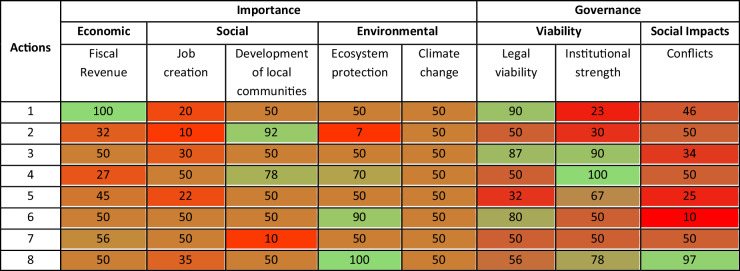
Example of an evaluation matrix for actions vs. importance and governance criteria

### Selection of the Most Relevant Uncertainties (Step 5)

**Fig. 4:**
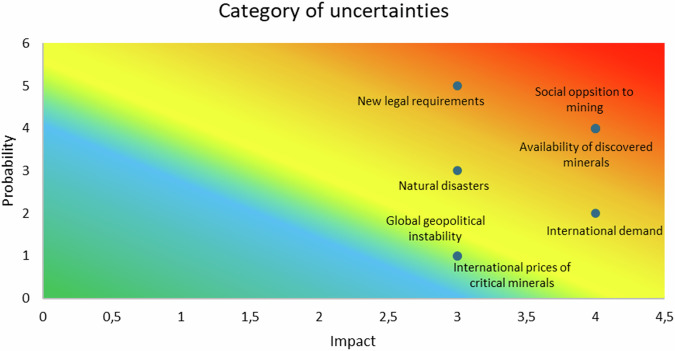
Heatmap result for uncertainty prioritization

The impact and probability assessments were initially pre-filled by the academic team and later reviewed by the UPME expert team. As an example, the uncertainty related to new legal requirements, considered a potential threat, was evaluated by experts as having a moderate impact but an almost certain probability of occurrence. According to the classification rules in Table 5, this combination results in an extreme priority ranking. Consequently, this uncertainty appears in the upper right quadrant of Fig. 4, within the red zone, indicating its high relevance for further analysis.

Another example is the uncertainty regarding natural disasters, specifically severe landslides, floods, or earthquakes that could impact the mining sector. Expert assessments determined that the expected negative impact would be moderate, as these events tend to be highly localized and would not disrupt the entire mining industry. Additionally, the probability of occurrence was rated as possible. Based on the association rules in Table 2, this moderate–possible combination classifies this uncertainty as high priority rather than extreme.

Following this exercise, only uncertainties classified as extreme priority were selected for sensitivity and robustness analysis. These include new legal requirements, mineral availability, armed conflicts, and social conflicts. These uncertainties are consistently considered throughout the prioritization exercise, but their robustness analysis is conducted separately for each strategic line and action. This step is essential for evaluating the sensitivity and robustness of the actions against extreme uncertainties, which will be further analyzed in the next stage.

### Robustness and Sensitivity Evaluation of Each Action Against Relevant Uncertainties (Step 6)

**Fig. 5:**
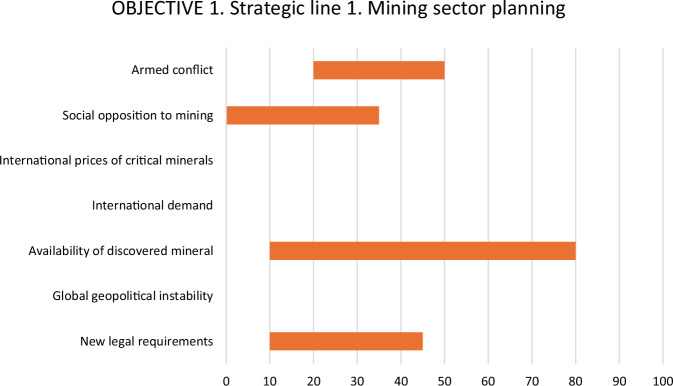
Tornado chart for Objective 1, Strategic Line 1 – Mining Planning

The robustness and sensitivity analysis provides a critical lens through which to evaluate how each action may perform under conditions of extreme uncertainty. This step ensures that prioritized actions are not only strategically relevant but also resilient across a range of plausible future scenarios. The results for Objective 1, Strategic Line 1 (Mining Sector Planning) are illustrated in Fig. 5 using a tornado chart. This visualization compares the sensitivity and robustness of each action with respect to key uncertainties. In this representation, the length of each bar indicates sensitivity—that is, the degree of fluctuation in an action’s performance between the best- and worst-case scenarios—while the portion of the bar above the predefined success threshold reflects robustness, i.e., the action’s capacity to remain effective despite adverse conditions.

Figure 5 illustrates the sensitivity and robustness of strategic actions to key uncertainty variables. The length of each bar represents the variation in expected performance between the best- and worst-case scenarios (sensitivity), while the portion of the bar above the predefined success threshold (typically 40%), defined as the minimum acceptable level of performance based on expert judgment, indicates the robustness of each action in the face of uncertainty. This analysis supports the identification of actions requiring enhanced monitoring and control measures.

To complement this analysis, a detailed robustness evaluation for Objectives 1, 2, 3, and 4 is included in Appendix B. This appendix provides further insights into how strategic actions behave when exposed to critical uncertainties, contributing to a more comprehensive understanding of their long-term viability.

In addition to the visual representation, this step integrates a qualitative evaluation approach grounded in expert judgment—particularly useful in contexts where fully developed quantitative models are unavailable. Experts assessed how each action would contribute to its strategic objective under the worst- and best-case realizations of each extreme-priority uncertainty. For example, in the case of Strategic Line 1 under Objective 1, the uncertainty “new legal requirements” was found to have a significant influence. Under the worst-case scenario, the estimated achievement level was 10%, while under the best-case scenario it reached 45%, resulting in a sensitivity of 35%. Since the best-case scenario barely exceeds the success threshold (40%), the robustness of this action to that uncertainty is only 5%.

This analysis not only quantifies the vulnerability of actions but also highlights where additional safeguards or institutional support may be required. Furthermore, the robustness matrix developed in this step complements the importance and governance matrices, forming a triad that supports a more comprehensive and defensible prioritization framework. By integrating these three dimensions—strategic relevance, governance feasibility, and resilience to uncertainty—decision-makers are better equipped to select actions that are both impactful and implementable under a wide range of future conditions.

### IGOR Multi-criteria Valuation of Each Action (Step 7)

The results of the IGOR multi-criteria evaluation provide a robust basis for prioritizing actions within each strategic line of the mining sector policy framework. By integrating the dimensions of Importance, Governance feasibility, and Robustness, the IGOR index enables a comparative analysis that highlights the actions with the greatest potential impact, implementability, and resilience under uncertainty.

For each action, the evaluation began by calculating scores for the three macrocriteria. The Importance score was derived from weighted averages across economic, social, and environmental criteria. For example, in the case of an action called *A1_1_1—Design and implement a strategy to integrate social and environmental variables into mining planning tools, in accordance with the institutional mandate—* the individual scores for criteria such as fiscal revenue, job creation, and environmental protection were weighted according to their assigned priority levels. This produced a normalized Importance score of 72.14, indicating high alignment with development priorities. Similarly, the *Governance score* for the same action was calculated based on the institutional capacity, feasibility, and risk of social conflict, yielding a high score of 89.44. The *Robustness score*, however, was more moderate (35%), reflecting vulnerabilities identified in Step 6, particularly with respect to uncertainties such as new legal requirements and social opposition to mining.

Using equal weights (0.33) for the three macrocriteria, the resulting IGOR index for action A1_1_1^1^ was 65.53%, placing it in the upper-middle range among the thirteen actions evaluated under Strategic Line 1 of Objective 1. Although the action demonstrated high strategic relevance and institutional feasibility, its moderate robustness suggests the need for close monitoring of its performance under adverse conditions. Other actions in the same strategic line exhibited different profiles: some showed lower importance but higher robustness, while others were limited by governance constraints despite strong contributions to strategic objectives.

These differences allowed for the generation of a prioritized ranking of actions within each strategic line. Actions with the highest IGOR scores emerged as top candidates for immediate implementation, while those with lower scores may require refinement, sequencing, or additional support mechanisms before execution. This disaggregated evaluation not only supports decision-making by highlighting high-impact actions but also allows for a more nuanced understanding of trade-offs between ambition, feasibility, and resilience.

A full ranking of actions, disaggregated by strategic objective and line, is presented in Appendix C. This appendix includes the individual scores for Importance, Governance, and Robustness, as well as the resulting IGOR index for each action. By structuring the results by objective and strategic line, Appendix C provides a detailed view of how each action performs across the evaluation dimensions, supporting a more transparent and systematic decision-making process. This information serves as a practical reference for prioritization, sequencing, and resource allocation in the implementation of mining sector policies.

Overall, the IGOR valuation consolidates the multi-dimensional prioritization process by translating complex and sometimes conflicting criteria into an actionable metric. This step closes the evaluation cycle, offering a comprehensive and replicable framework for prioritizing public policies in complex sectors such as mining, where uncertainty and trade-offs are inherent in strategic decision-making.

### Sensitivity Analysis (Step 8)

The results of the sensitivity analysis confirm the robustness of the prioritization obtained through the IGOR index. For each action, 200 simulations were conducted, each using a different set of randomly generated weights for the three macrocriteria. The priority rank of each action was recalculated in every iteration, producing a frequency distribution of its position within the hierarchy.

In the Colombian case study, the original macro-criteria weights were defined by a technically qualified group composed of members of the UPME Mining Subdirectorate and academic researchers involved in the development of the NMDP. These experts have experience in mining policy, economic evaluation, sustainability assessment, and multicriteria decision analysis. The Monte Carlo simulation therefore evaluates how the priorization would vary under alternative weighting configurations beyond the preferences initially expressed by this expert group.

**Fig. 6:**
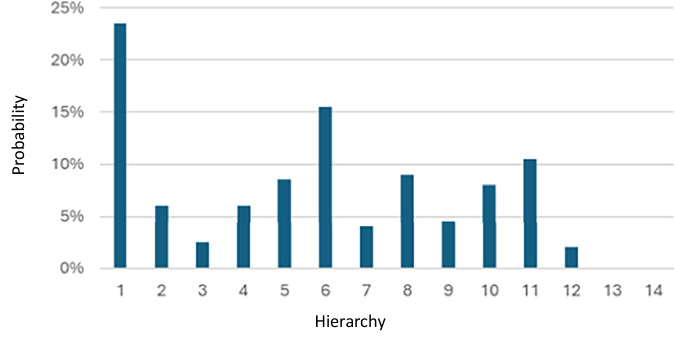
Example of priority ranking histogram for Action A1_1_1

For illustrative purposes, Fig. 6 shows the histogram of priority rankings for action A1_1_1, which had an original position of 4 within Strategic Line 1 of Objective 1. The simulated rankings varied across the iterations, but the distribution remained concentrated around the mid-range values. The mean rank observed over the 200 simulations was 5, indicating only a minor deviation from the initial position. This outcome suggests that the action’s prioritization is relatively stable, even when subjected to significant variation in the weights assigned to the evaluation dimensions.

The prioritization positions for all actions across each strategic line and objective can be consulted in Appendix C, which provides a complete overview of the IGOR scores and their corresponding ranks. By comparing the mean ranks and frequency distributions generated through sensitivity analysis to the original hierarchy in Appendix C, decision-makers can better understand which actions are consistently prioritized and which ones are more sensitive to preference changes.

These findings enhance the analytical depth of the IGOR methodology by adding a layer of probabilistic validation to the prioritization results. The inclusion of this simulation-based perspective supports more robust policy formulation and strengthens the legitimacy of decisions under uncertainty.

## Conclusions

This study presents the IGOR methodology as an innovative and integrative approach for prioritizing public policy actions in complex and uncertain decision-making environments. By combining the strengths of four well-established tools—Importance-Governance Matrix (IGM), Analytic Hierarchy Process (AHP), Tornado analysis, and Monte Carlo simulation—IGOR addresses key limitations of traditional prioritization techniques, offering a comprehensive framework that explicitly incorporates uncertainty, stakeholder preferences, and multidimensional evaluation.

The application of IGOR to *2024 - 2035 Colombia’s National Mining Development Plan* illustrates its practical value in supporting strategic planning processes. The selection of evaluation criteria emerged from an iterative and collaborative dialogue between academic researchers and technical experts from UPME, ensuring alignment with sectoral priorities and institutional realities. Despite the early stage of policy development and the limited availability of data, the methodology enabled a rigorous and structured prioritization of over 100 proposed actions. To reduce expert burden, only the most impactful criteria were retained, balancing analytical depth with cognitive feasibility.

The IGOR index integrates three macrocriteria—Importance, Governance, and Robustness—into a single prioritization score. These scores are grounded in expert judgment and validated through Monte Carlo-based sensitivity analysis, which tested 200 random weight configurations per action. The resulting stability of action rankings across simulations reinforces confidence in the prioritization outcomes and enhances their legitimacy for decision-makers. Importantly, IGOR is designed as a decision support tool—not a prescriptive ranking mechanism. It provides mining authorities with a set of structured metrics and analytical procedures to inform final decisions, even in contexts of uncertainty, incomplete information, or evolving priorities.

Beyond these quantitative results, the application of IGOR generated relevant analytical insights that strengthen its contribution beyond methodological demonstration. From the UPME perspective, the method was perceived as accessible and useful for supporting decision-making under uncertainty, facilitating structured discussions that integrate economic, social, and environmental dimensions through intuitive and scenario-based reasoning. More importantly, the application revealed that key policy concepts—such as importance and governance—are inherently multidimensional and dependent on stakeholder perspectives and future conditions, rather than fixed or aggregated indicators. Its implementation did not require specialized software, which enhanced its usability in institutional settings. From the academic perspective, the method proved to be implementable and flexible, although it required careful attention to the elicitation and representation of stakeholder preferences. Notably, the process revealed non-intuitive trade-offs—particularly within social dimensions—and enabled the identification of priority actions that may not have emerged through conventional approaches. While minor adjustments were made during implementation, these did not alter the core structure of the methodology, confirming its adaptability. Although the application of IGOR requires a certain level of expertise, this is consistent with the demands of multi-criteria decision-making in complex policy contexts rather than a limitation of the approach.

From a methodological standpoint, IGOR offers a replicable and adaptable framework for strategic prioritization in public policy. Its integration of participatory inputs with formal analytical methods enables a more transparent, resilient, and evidence-informed planning process. From an applied perspective, it delivers concrete guidance for the Colombian mining sector and serves as a prototype for other sectors where decisions must be made under uncertainty and limited data. In conclusion, IGOR supports more informed, inclusive, and robust decision-making processes. Its hybrid architecture—grounded in expert knowledge and probabilistic validation—can be adapted to diverse governance challenges beyond mining. Finally, further research may focus on expanding stakeholder engagement, incorporating real-time data as it becomes available, and tailoring the methodology to new domains facing the urgent need for prioritization under uncertainty.

## Supplementary information

APPENDIX A

APPENDIX B

APPENDIX C

Supplementary Material_1

Supplementary Material_2

## Footnotes

1 The code A1_1_1 refers to a specific action within the evaluation framework: the letter “A” stands for “Action”; the first number (1) indicates that it belongs to Objective 1; the second number (1) denotes its corresponding strategic line within that objective; and the final number (1) identifies the action number within that strategic line. All actions within each strategic line were sequentially numbered for consistency and traceability.
