# Supplementary material for: Strategic Prioritization of Mining Policies in Colombia Through The IGOR Hybrid Framework
Source: Environ Manage. 2026 Jun 11;76(6):213. doi: 10.1007/s00267-026-02500-6 (PMC13260195; doi:10.1007/s00267-026-02500-6)
Supplement: Supplementary file 1 — APPENDIX A [file 267_2026_2500_MOESM1_ESM.docx]

**APPENDIX A. FULL LIST OF IDENTIFIED ACTIONS FOR PRIORITIZATION**

| **Strategic Objective** | **Strategic Line** | **Actions** |  |
| --- | --- | --- | --- |
|  |  |  |  |
| **Objective 1.**  **Strengthen mining activity planning with social, cultural, environmental, and territorial approaches for the rational use of mineral resources.** | 1. **Mining Sector Planning**   Develop mining planning as a management tool to support decision-making and achieve greater efficiency, effectiveness, and quality in the rational use of resources through an environmental, social, and cultural approach. | Design and implement a strategy for integrating social and environmental variables into mining planning tools in accordance with the institution's mission. |  |
|  |  | Implement a strategy to integrate a gender perspective into mining sector planning tools. |  |
|  |  | Update the National Energy Plan to include strategic minerals for the country, as determined by the National Mining Agency. |  |
|  |  | Develop the sub-sectoral plan for minerals for a just energy transition. |  |
|  |  | Develop the sub-sectoral plan for minerals for food security. |  |
|  |  | Develop the sub-sectoral plan for minerals for infrastructure and habitat. |  |
|  |  | Implement a methodology for formulating planning instruments in the mining sector. |  |
|  |  | Delimit and declare Strategic Mining Areas (EMAs) and Strategic Mining Areas for Formalization (EMA-F). |  |
|  |  | Determine the geological and mineral potential of new areas under evaluation, as technical support for establishing strategies, plans, programs, and projects for managing strategic and critical minerals. |  |
|  |  | Conduct metallogenic studies at the national level as technical support for establishing strategies, plans, programs, and projects for managing strategic and critical minerals. |  |
|  |  | Increase knowledge of mineral resources, their potential, and interactions with the environment and human health as technical support to promote environmentally and socially responsible strategies for managing strategic and critical minerals in the national territory. |  |
|  |  | Conduct cartographic and geomorphological studies. |  |
|  |  | Generate geoscientific knowledge through research, evaluation, and monitoring of geologically-originated hazards as a basis for comprehensive risk management and sustainable development in the country. |  |
|  | 1. **Territorial Planning**   Measures for Mining Activity Develop strategies that promote territorial planning in mining areas to foster harmonious relationships in territories and contribute to resolving existing issues. | Develop tools for the delimitation and implementation of Mining Districts for productive diversification, including diagnostic documents, methodologies, strategic plans, among others. |  |
|  |  | Structure strategic management plans within the framework of the delimitation of Mining Districts for productive diversification. |  |
|  |  | Approve strategic management plans within the framework of the delimitation of Mining Districts for productive diversification. |  |
|  |  | Update management plans according to the characteristics and needs of each delimited mining district. |  |
|  |  | Develop tools to include mining-related topics in municipal and departmental land-use planning (POT, PBOT, EOT). |  |
|  |  | Design and implement an educational strategy to guide municipal mayors, community councils, and Indigenous authorities on land-use planning around mining activity. |  |
|  |  | Conduct a climate change risk analysis for the mining sub-sector to identify climate change adaptation needs. |  |
|  |  | Advance the implementation of mining substitution programs, including closure, dismantling, restoration, and reshaping of areas affected by mining activities in designated páramo ecosystems. |  |
|  | 1. **Conflict Mitigation and Incorporation of Human Rights, Ethnic, Gender, and Intersectional Approaches in Mining Activity**   Promote the well-being of communities and territories where mining activity occurs through dialogue, participation, conflict management, the recognition of ethnic communities, and the inclusion of gender, differential, and intersectional approaches. | Create dialogue spaces to strengthen the institutional presence of the National Mining Agency in territories. |  |
|  |  | Implement the territorial engagement program. |  |
|  |  | Promote social appropriation of geoscientific and nuclear knowledge to contribute to the country’s social, environmental, and economic well-being. |  |
|  |  | Implement a strategy to strengthen the territorial and ethnic focus and presence in mining planning. |  |
|  |  | Monitor conflicts in the mining sector through the conflict observatory. |  |
|  |  | Consolidate social and socio-environmental conflict data from various ANM processes into the ANM relationship observatory. |  |
|  |  | Design an educational strategy to guide the formulation and monitoring of social management plans in mining activity |  |
|  |  | Develop tools to monitor and evaluate the implementation of social management plans in mining activity. |  |
|  |  | Monitor the human rights policy in the mining-energy sector. |  |
|  |  | Monitor the gender policy in the mining-energy sector. |  |
| **Objective 2.**  **Strengthen the institutional framework of mining activity to improve governance, transparency, excellence in processes, the streamlining of procedures, and oversight, while promoting institutional communication and engagement.** | 1. **Institutional Governance of the Mining Sector**   Strengthen institutional governance and the mining sector through the promotion of tools and technical instruments that enhance operational dynamics and environmental best practices, in order to consolidate a regulatory framework focused on the rational use of mineral resources, productivity, and formalization. | Establish a roadmap for the unification and modernization of the mining sector's regulatory framework. |  |
|  |  | Develop strategies to improve response times in administrative procedures that facilitate the materialization of new mining developments associated with strategic minerals. |  |
|  |  | Establish territorial dialogue spaces through inter-institutional coordination to design work plans for the formalization of small-scale mining. |  |
|  |  | Implement supervision and audit mechanisms to monitor the execution of actions outlined in mine closure and abandonment plans, ensuring their compliance. |  |
|  |  | Incorporate and/or update information on temporary reserves in the Integrated Mining Management System (SIGM) to guarantee traceability and efficient resource and reserve management. |  |
|  |  | Develop a portfolio of climate change adaptation measures for the mining sector in alignment with action lines identified in risk assessments |  |
|  |  | Optimize and continuously improve the AnnA-Minería management platform to facilitate the handling of mining information and the development of various procedures under its jurisdiction. |  |
|  | 1. **Interinstitutional Collaboration and Coordination**   Promote effective coordination mechanisms that define roles, establish common protocols to enable interoperability between information systems, and foster efficient collaboration for the secure and timely exchange of data and inputs that facilitate institutional coordination**.** | Stimulate and manage the Mining Planning Coordination Committee (COCPMI) as a permanent communication and coordination platform among mining sector entities and their interaction with other institutions. |  |
|  |  | Establish strategic public-private partnerships for specific mining sector projects. |  |
|  |  | Generate strategic alliances between the public and private sectors for specific projects in small-scale and subsistence mining. |  |
|  |  | Promote effective coordination and collaboration mechanisms between the National Mining Agency (ANM) and the National Disaster Risk Management Unit (UNGRD) for risk prevention in mining activities. |  |
|  |  | Establish effective coordination and collaboration mechanisms for information exchange, preventive actions, and traceability control, as well as for detecting unauthorized and illicit mining activities. |  |
|  |  | Coordinate actions to strengthen professionalization, workforce competency development, and training on mining safety for personnel involved in mining activities. |  |
|  |  | Strengthen the Colombian Mining Information System (SIMCO) through the updating of mining sector data to serve as inputs for the analysis unit that supports decision-making and policy development. |  |
|  | 1. **Institutional Communication and Engagement**   Strengthen the relationship and communication between the community and mining institutions, mining companies and their work teams, ensuring transparency and efficiency in interaction and communication processes, while positioning and improving the image of the sector. | Establish interinstitutional working groups in mining districts designated by the Ministry of Mines and Energy (MME) for the analysis of social, environmental, mining, territorial, and other relevant components. |  |
|  |  | Develop communication strategies to raise public awareness and visibility regarding the Ministry’s management efforts, the transformation of Colombia's mining model, and the just energy transition. |  |
|  |  | Periodically produce sectoral bulletins with relevant information about mining activity during the reporting period, to promote a comprehensive understanding of Colombia’s mining sector. |  |
|  |  | Improve and enhance the institutional image of the sector through communication strategies. |  |
|  |  | Create training and advisory spaces on mechanisms for citizen participation, legal updates, requirements, procedures, and the roles of institutions in mining matters. |  |
| **Objective 3.**  **Promote and encourage the regularization of mining and the implementation of more efficient extraction, processing, and transformation techniques and technologies, to strengthen the sector and address the challenges of mining activity, including safety, minimization of environmental impacts, ecosystem rehabilitation and/or recovery, and community well-being.** | 1. **Mining Formalization**   Implement actions aimed at promoting, encouraging, and supporting access to legality for small-scale and subsistence mining across the national territory, through processes such as associativity and mining formalization, among others. | Provide technical assistance to beneficiary Mining Production Units (UPMs) based on their vocation and formalization path. |  |
|  |  | Develop a strategy for establishing mediation processes to reach agreements between mining title holders and informal small-scale miners. |  |
|  |  | Implement actions from the Unified Plan for Legalization and Formalization of Mining (PULF) to facilitate access to formalization for small-scale mining. |  |
|  |  | Provide mechanisms that allow for the identification, registration, and targeting of subsistence (artisanal) miners to protect their activity under dignified conditions. |  |
|  |  | Design and implement a strategy to strengthen knowledge and facilitate understanding of formalization and/or titling procedures for mining activities in the territories. |  |
|  |  | Assist informal, traditional, and small-scale miners in the process of formalization or in their transition to any formalization figure, especially regarding the creation of associative schemes. |  |
|  |  | Implement communication and training strategies to strengthen knowledge and facilitate understanding of administrative procedures related to Special Reserve Areas as a mechanism for formalization. |  |
|  |  | Implement communication, training, and engagement strategies in and with the territories to strengthen knowledge and facilitate understanding of formalization and/or titling procedures for mining activities. |  |
|  | 1. **Mining Safety**   Strengthen risk prevention and management in mining activities and enhance response capacity to mining emergencies. | Design and implement a periodic reporting mechanism that provides structured information on non-compliance in mining safety identified in the field. |  |
|  |  | Design and implement a tool to monitor compliance with mining safety measures and the implementation of improvement plans for their resolution or closure. |  |
|  |  | Train personnel in standards for prevention, mining safety, accident investigation methodologies, and emergency response. |  |
|  |  | Design guides, protocols, or practical tools to identify and manage risks inherent to mining activities and disseminate them among workers. |  |
|  |  | Design the Behavioral Change Management Plan in Mining Safety. |  |
|  |  | Implement previously designed behavioral change management plans in Mining Safety. |  |
|  |  | Strengthen training strategies for emergency preparedness and response in the framework of Disaster Risk Management. |  |
|  |  | Implement the National Mining Safety Policy. |  |
|  |  | Carry out the adaptation, maintenance, and continuous improvement of mining rescue facilities. |  |
|  |  | Acquire and maintain specialized mining rescue equipment. |  |
|  | 1. **Quality and Efficiency (Good Practices)**   Strengthen activities across the mining cycle through technical assistance, training, financing, and the implementation of good practices and improved techniques, with an integrated vision that includes the environment, community, and territory. | Promote the creation and/or strengthening of a network of technology development centers and science, technology, and innovation parks for the mining sector. |  |
|  |  | Provide comprehensive technical assistance to traditional and small-scale miners to support the consolidation of viable and sustainable mining projects. |  |
|  |  | Establish guidelines that promote the responsible development of subsistence (artisanal) mining, considering social, cultural, and environmental factors. |  |
|  |  | Encourage the application of geological-mining, social, and environmental best practices in small-scale mining activities, aligned with the technical and regulatory instruments developed for small-scale mining. |  |
|  |  | Develop methodological guidelines for the sustainable use of strategic and critical minerals without the use of polluting substances (technical guidelines on socially and environmentally manageable mineral beneficiation). |  |
|  |  | Adopt environmental mining guides for small-scale mining and formalization figures. |  |
|  |  | Disseminate updated environmental mining guides for exploration, exploitation, processing, beneficiation, formalization, and small-scale mining, to promote environmentally sound practices at all territorial mining scales. |  |
|  |  | Provide support to territorial entities and/or mining communities for the development of initiatives aimed at improving mining practices. |  |
|  |  | Promote best practices in mining management and in relationships with communities. |  |
|  |  | Analyze information related to mercury contamination and other pollutants from mining activities. |  |
|  |  | Monitor and evaluate the National Action Plan on mercury in artisanal and small-scale mining in Colombia. |  |
|  |  | Implement a strategy to monitor "due diligence in the supply chain" processes for mining title holders. |  |
|  | 1. **Promotion of Innovation and Technology Use**   Promote research, development, and implementation of cleaner and more efficient technologies in mineral exploration, extraction, and processing, as well as encourage circular economy models and the adoption of alternative energies to achieve greater energy efficiency and reduce ecosystem impacts and/or contribute to their rehabilitation and recovery. | Create and strengthen a digital mineral fingerprinting and/or traceability tool for determining origin and illicit substance use in beneficiation processes. |  |
|  |  | Strengthen and consolidate technical expertise and a network of laboratories for material characterization of geological interest to generate geoscientific and nuclear knowledge in the country. |  |
|  |  | Implement strategies to adopt clean and cutting-edge mining practices through technological transfer in territories to reduce carbon footprints and enhance competitiveness. |  |
|  |  | Structure and implement work plans to promote energy efficiency and self-generation in titled mining areas, in line with the roadmap for a just energy transition. |  |
|  |  | Implement strategies to promote the adoption of quality standards that increase added value throughout the mineral production chain. |  |
| **Objective 4.**  **Strengthen productive transformation to consolidate value chains based on the responsible exploitation of strategic minerals and establish new activities that generate added value.** | 1. **Productive Linkages**   Develop productive linkages between regions and sectors of the Colombian economy based on the exploitation of strategic minerals. | Develop coordination spaces that promote the strengthening and development of value chains that add value to strategic minerals for reindustrialization, energy transition, agricultural development, and public infrastructure. |  |
|  |  | Implement a strategy of agglomerations or productive clusters to create linkages in mining activities across sectors and regions. |  |
|  |  | Create a document integrating strategies for developing value-added industries in territories based on strategic minerals. |  |
|  |  | Develop strategies to strengthen value chains that add value to strategic minerals for reindustrialization, energy transition, food sovereignty, and public infrastructure. |  |
|  |  | Identify and strengthen mining agglomerations and supply chains with a vision focused on reindustrialization. |  |
|  |  | Formulate and implement projects focused on energy efficiency, self-generation, productive diversification, and/or reindustrialization in mining-oriented regions. |  |
|  |  | Build a roadmap for the promotion of sustainable productive projects in companies involved in mining value chains that incorporate circular economy, bioeconomy, resource efficiency, and climate change management, among others. |  |
|  |  | Implement strategies to bridge the supply and demand of a prioritized mineral. |  |
|  |  | Design and implement strategies to attract, retain, identify, and facilitate the reception of sustainable foreign direct investment (FDI) and technology transfer for the development of productive linkages and the reindustrialization of the country based on strategic minerals. |  |
|  |  | Identify, through specialized sectoral studies, possible bottlenecks or inefficiencies in value chains in primary and secondary economic sectors related to the mining industry. |  |
|  |  | Evaluate sub-sectoral plans for energy transition, food security, infrastructure, and habitat. |  |
|  | **2.Labor Conversion and Diversification**  Design and implement strategies for labor conversion and productive diversification that involve mining populations and their families, based on innovation, access to technology, and financing. | Prepare an annual report consolidating the quantification of energy generation by Non-Conventional Renewable Energy Sources (FNCER) contributed by the mining sector. |  |
|  |  | Develop a strategy to promote the use of FNCER and alternative low-greenhouse gas (GHG) emission energy sources in the mining sector. |  |
|  |  | Implement a program for the labor reconversion or relocation of small-scale, artisanal, and formalized miners, including those in the PULF mechanism. |  |
|  |  | Implement a strategy to strengthen productive and employment alternatives in mining-oriented territories. |  |
|  |  | Manage the development of reconversion and productive diversification alternatives for subsistence (artisanal), small-scale, and traditional miners. |  |
|  |  | Implement a socio-business development program to promote financial inclusion and the creation and/or strengthening of associative work schemes, also generating value-added opportunities in small-scale mining. |  |
|  |  | Formulate and implement a program to strengthen and identify financing sources for socio-business actors, underserved, and unserved populations. |  |
