# Supplementary material for: Strategic Prioritization of Mining Policies in Colombia Through The IGOR Hybrid Framework
Source: Environ Manage. 2026 Jun 11;76(6):213. doi: 10.1007/s00267-026-02500-6 (PMC13260195; doi:10.1007/s00267-026-02500-6)

**APPENDIX B. ROBUSTNESS AND SENSITIVITY ANALYSIS OF STRATEGIC ACTIONS UNDER EXTREME UNCERTAINTIES**


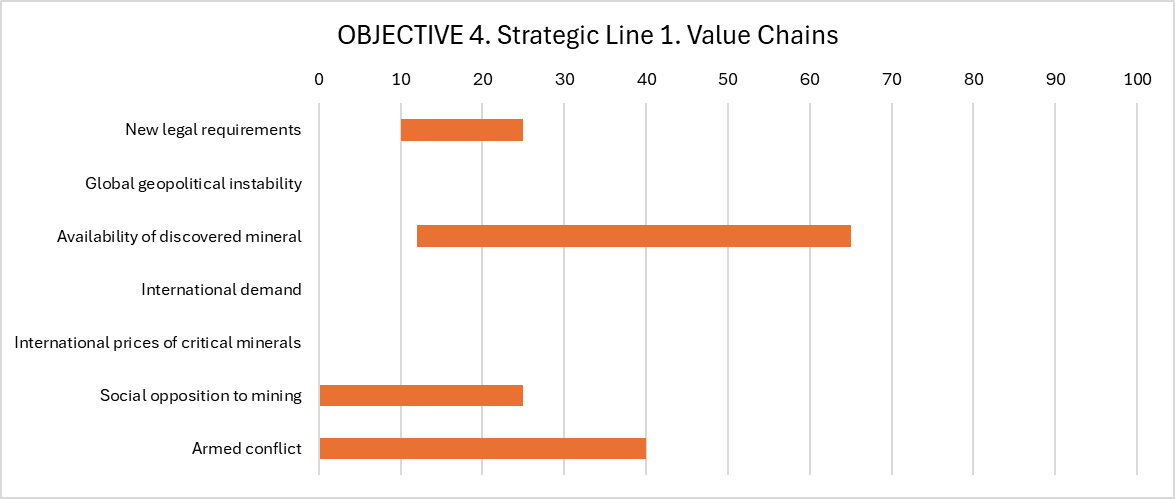


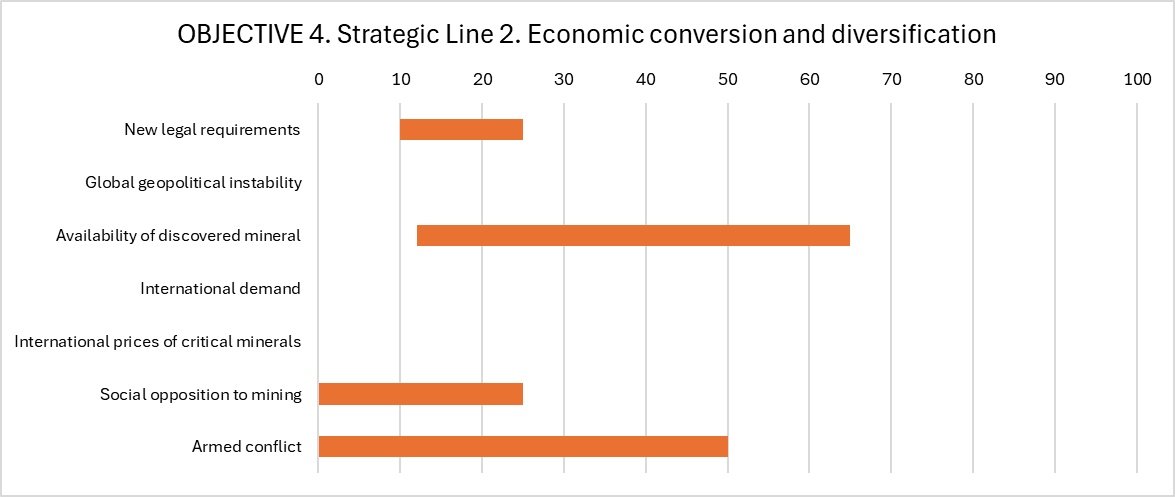

Supplement: Supplementary file 2 — APPENDIX B [file 267_2026_2500_MOESM2_ESM.docx]
