# Supplementary material for: Strategic Prioritization of Mining Policies in Colombia Through The IGOR Hybrid Framework
Source: Environ Manage. 2026 Jun 11;76(6):213. doi: 10.1007/s00267-026-02500-6 (PMC13260195; doi:10.1007/s00267-026-02500-6)
Supplement: Supplementary file 3 — APPENDIX C [file 267_2026_2500_MOESM3_ESM.docx]

**APPENDIX C. IGOR Index Scores and Prioritization of Actions across all Strategic Objectives and Lines**

- Objective 1Strategic Line 1:

| **Acción** | **ID Acción OBJ_LINEA_ACCION** | **Importancia** | **Gobernabilidad** | **Robustez** | **Categoria importancia** | **Categoria Gobernabilidad** | **Categoria Acción** | **AHP total** | **Prioridad** |
| --- | --- | --- | --- | --- | --- | --- | --- | --- | --- |
| Diseñar e implementar una estrategia de integración de variables sociales y ambientales en las herramientas de planeación minera conforme la misionalidad de la entidad. | **A1_1_1** | 72,14 | 89,44 | 35,00 | Alta | Alta | I:Urgente | 65,53 | 4 |
| Implementar una estrategia de integración de la perspectiva de género en las herramientas de planeación minera. | **A1_1_2** | 22,14 | 54,44 | 55,00 | Baja | Alta | III:Menos  Urgente | 43,86 | 13 |
| Actualizar en el Plan Energético Nacional los minerales estratégicos para el país, determinados por la Agencia Nacional de Minería. | **A1_1_3** | 60,71 | 47,78 | 87,50 | Alta | Baja | II:Retadora | 65,33 | 5 |
| Elaborar el plan subsectorial de minerales para la transición energética justa | **A1_1_4** | 62,86 | 66,11 | 87,50 | Alta | Alta | I:Urgente | 72,16 | 1 |
| Elaborar el plan subsectorial de minerales para la seguridad alimentaria | **A1_1_5** | 54,29 | 66,11 | 87,50 | Alta | Alta | I:Urgente | 69,30 | 2 |
| Elaborar el plan subsectorial de minerales para la infraestructura y hábitat | **A1_1_6** | 54,29 | 66,11 | 87,50 | Alta | Alta | I:Urgente | 69,30 | 2 |
| Implementar una metodología para la formulación de instrumentos de planeación del sector minero. | **A1_1_7** | 42,14 | 73,33 | 55,00 | Baja | Alta | III:Menos  Urgente | 56,83 | 8 |
| Delimitar y declarar Áreas Estratégicas Mineras (AEM) y Áreas Estratégicas Mineras para la Formalización (AEMF) | **A1_1_8** | 61,43 | 86,67 | 22,50 | Alta | Alta | I:Urgente | 56,87 | 7 |
| Determinar el potencial geológico y mineral de las nuevas áreas sujetas a evaluación, como soporte técnico para el establecimiento de estrategias, planes, programas y proyectos de gestión de minerales estratégicos y críticos. | **A1_1_9** | 28,57 | 47,78 | 87,50 | Baja | Baja | IV:puede  esperar | 54,62 | 9 |
| Realizar estudios metalogénicos en el territorio nacional como soporte técnico para el establecimiento de estrategias, planes, programas y proyectos de gestión de minerales estratégicos y críticos. | **A1_1_10** | 25,00 | 47,78 | 87,50 | Baja | Baja | IV:puede  esperar | 53,43 | 10 |
| Aumentar el conocimiento de los recursos minerales, su potencial e interacción con el medio ambiente y la salud humana como soporte técnico para la promover estrategias ambiental y socialmente responsables de gestión de minerales estratégicos y críticos del territorio nacional. | **A1_1_11** | 31,79 | 63,33 | 63,75 | Baja | Alta | III:Menos  Urgente | 52,96 | 11 |
| Realizar estudios cartográficos y geomorfológicos | **A1_1_12** | 43,57 | 61,11 | 87,50 | Baja | Alta | III:Menos  Urgente | 64,06 | 6 |
| Generar conocimiento geocientífico mediante la investigación, evaluación y monitoreo de las amenazas de origen geológico, como base para la gestión integral del riesgo y el desarrollo sostenible del país. | **A1_1_13** | 35,71 | 47,78 | 63,75 | Baja | Baja | IV:puede  esperar | 49,08 | 12 |

- Objective 1 Strategic Line 2

| **Acción** | **ID Acción OBJ_LINEA_ACCION** | **Importancia** | **Gobernabilidad** | **Robustez** | **Categoria importancia** | **Categoria Gobernabilidad** | **Categoria Acción** | **AHP total** | **Prioridad** |
| --- | --- | --- | --- | --- | --- | --- | --- | --- | --- |
| Desarrollar herramientas para la delimitación y puesta en marcha de los Distritos Mineros para la diversificación productiva entre otros a traves de documentos diagnósticos, metodologías, planes estratégicos entre otros. | **A1_2_1** | 60,71 | 88,89 | 26,25 | Alta | Alta | I:Urgente | 58,62 | 3 |
| Elaborar actos administrativos que permitan delimitar y declarar los Distritos Mineros para la diversificación productiva | **A1_2_2** | 57,14 | 88,89 | 80,00 | Alta | Alta | I:Urgente | 75,34 | 1 |
| Desarrollar herramientas que permitan incluir en los planes de ordenamiento territorial (POT, PBOT, EOT) municipales y departamentales lo relacionado con el sector minero. | **A1_2_3** | 61,07 | 79,44 | 80,00 | Alta | Alta | I:Urgente | 73,51 | 2 |
| Diseñar e implementar una estrategia pedagógica para orientar a las alcaldías municipales, consejos comunitarios y autoridades indígenas, sobre el ordenamiento territorial en torno a la actividad minera. | **A1_2_4** | 29,29 | 81,11 | 26,25 | Baja | Alta | III:Menos  Urgente | 45,55 | 7 |
| Elaborar el análisis de riesgo por cambio climático para el subsector de minería para la identificación de necesidades de adaptación al cambio climático | **A1_2_5** | 45,36 | 45,56 | 62,50 | Baja | Baja | IV:puede  esperar | 51,14 | 6 |
| Avanzar en la implementación de los programas de sustitución de actividades mineras que incluyen las actividades de cierre, desmantelamiento, restauración y reconformación de las áreas intervenidas por las actividades mineras en páramos delimitados | **A1_2_6** | 86,43 | 42,22 | 26,25 | Alta | Baja | II:Retadora | 51,63 | 5 |
| Avanzar en la implementación y apropiación de la Politica de Gestión del Riesgo de Desastres del Sector del Minero Energetico (Resolución 40411 de 2021), mediante la consideración de riesgos tecnologicos. | **A1_2_7** | 56,43 | 91,11 | 26,25 | Alta | Alta | I:Urgente | 57,93 | 4 |

- Objective 1 Strategic Line 3

| **Acción** | **ID Acción OBJ_LINEA_ACCION** | **Importancia** | **Gobernabilidad** | **Robustez** | **Categoria importancia** | **Categoria Gobernabilidad** | **Categoria Acción** | **AHP total** | **Prioridad** |
| --- | --- | --- | --- | --- | --- | --- | --- | --- | --- |
| Generar espacios de diálogo para el fortalecimiento de la presencia institucional de la Agencia Nacional de Minería en los territorios. | **A1_3_1** | 36,79 | 83,33 | 72,50 | Baja | Alta | III:Menos  Urgente | 64,21 | 3 |
| Implementar el programa de relacionamiento con el territorio | **A1_3_2** | 33,93 | 82,22 | 60,00 | Baja | Alta | III:Menos  Urgente | 58,72 | 7 |
| Promover actividades de apropiación social del conocimiento geocientífico y nuclear para contribuir al bienestar social, ambiental y económico del país | **A1_3_3** | 36,07 | 70,00 | 72,50 | Baja | Alta | III:Menos  Urgente | 59,52 | 5 |
| Implementar una estrategia para el fortalecimiento del enfoque y presencia territorial- étnica para la planeación Minera | **A1_3_4** | 29,29 | 75,56 | 72,50 | Baja | Alta | III:Menos  Urgente | 59,11 | 6 |
| Realizar el seguimiento de las conflictividades del sector minero energético a través del observatorio de conflictividades. | **A1_3_5** | 46,43 | 67,78 | 72,50 | Baja | Alta | III:Menos  Urgente | 62,24 | 4 |
| Consolidar la información social y de conflictividad socioambiental desde diferentes procesos de la ANM, en el observatorio de relacionamiento de la ANM. | **A1_3_6** | 44,29 | 80,56 | 85,00 | Baja | Alta | III:Menos  Urgente | 69,95 | 1 |
| Diseñar una estrategia pedagógica para orientar la formulación y seguimiento de la elaboración de los planes de gestión social en la actividad minera. | **A1_3_7** | 42,14 | 80,00 | 85,00 | Baja | Alta | III:Menos  Urgente | 69,05 | 2 |
| Desarrollar herramientas para el seguimiento y evaluación de la implementación de los planes de gestión social en la actividad minera. | **A1_3_8** | 16,79 | 60,00 | 85,00 | Baja | Alta | III:Menos  Urgente | 53,93 | 9 |
| Realizar seguimiento a la política de Derechos Humanos del sector minero energético | **A1_3_9** | 11,43 | 65,56 | 72,50 | Baja | Alta | III:Menos  Urgente | 49,83 | 10 |
| Realizar seguimiento a la política de género del sector minero energético | **A1_3_10** | 11,43 | 68,89 | 85,00 | Baja | Alta | III:Menos  Urgente | 55,11 | 8 |

- Objective 2 Strategic Line 1

| **Acción** | **ID Acción OBJ_LINEA_ACCION** | **Importancia** | **Gobernabilidad** | **Robustez** | **Categoria importancia** | **Categoria Gobernabilidad** | **Categoria**  **Acción** | **AHP total** | **Prioridad** |
| --- | --- | --- | --- | --- | --- | --- | --- | --- | --- |
| Establecer una hoja de ruta para la unificación y modernización del marco regulatorio del sector minero | **A2_1_1** | 35,00 | 88,33 | 82,5 | Baja | Alta | III:Menos  Urgente | 68,61 | 2 |
| Generar estrategias para mejorar los tiempos de respuesta en los trámites que permitan la materialización de los nuevos desarrollos mineros asociados a los minerales estratégicos | **A2_1_2** | 30,71 | 66,67 | 82,5 | Baja | Alta | III:Menos  Urgente | 59,96 | 6 |
| Crear espacios de relacionamiento y articulación interinstitucional para mejorar los mecanismos y estrategias para la formalización minera. | **A2_1_3** | 55,36 | 91,67 | 100,0 | Alta | Alta | I:Urgente | 82,34 | 1 |
| Implementar mecanismos de supervisión y auditoría para fiscalizar la ejecución de las acciones contempladas en los planes de cierre y abandono, asegurando así su cumplimiento | **A2_1_4** | 33,21 | 80,00 | 82,5 | Baja | Alta | III:Menos  Urgente | 65,24 | 4 |
| Incorporar y/o actualizar la información sobre las reservas temporales en el Sistema Integral de Gestión Minera - SIGM, para garantizar la trazabilidad y gestión eficiente de los recursos y reservas | **A2_1_5** | 38,57 | 76,67 | 82,5 | Baja | Alta | III:Menos  Urgente | 65,91 | 3 |
| Elaborar un portafolio de medidas de adaptación al cambio climático para el sector minero, en relación con las líneas de acción identificadas en el análisis de riesgos | **A2_1_6** | 41,43 | 70,00 | 65,0 | Baja | Alta | III:Menos  Urgente | 58,81 | 7 |
| Implementación de las medidas de adaptación al cambio climático para el sector minero. | **A2_1_7** | 25,71 | 50,00 | 65,0 | Baja | Alta | III:Menos  Urgente | 46,90 | 8 |
| Optimizar y generar mejora continua en la plataforma de gestión AnnA-Minería para facilitar el manejo de la información minera y el desarrollo de los diferentes trámites de su competencia | **A2_1_8** | 53,21 | 51,67 | 82,5 | Alta | Alta | I:Urgente | 62,46 | 5 |

- Objective 2 Strategic Line 2

| **Acción** | **ID Acción OBJ_LINEA_ACCION** | **Importancia** | **Gobernabilidad** | **Robustez** | **Categoria importancia** | **Categoria Gobernabilidad** | **Categoria**  **Acción** | **AHP total** | **Prioridad** |
| --- | --- | --- | --- | --- | --- | --- | --- | --- | --- |
| Dinamizar y gestionar el Comité de Coordinación de Planeación Minera - COCPMI como instancia de comunicación y coordinación permanente entre las entidades pertenecientes al sector minero y su interrelación con las demás entidades | **A2_2_1** | 13,21 | 72,61 | 82,5 | Baja | Alta | III:Menos  Urgente | 56,11 | 4 |
| Pactar alianzas estratégicas entre el sector público y el privado para proyectos específicos del sector minero | **A2_2_2** | 39,29 | 47,39 | 82,5 | Baja | Baja | IV:puede  esperar | 56,39 | 3 |
| Promover mecanismos de coordinación y colaboración efectivos entre la Autoridad Minera - ANM y la Unidad Nacional de Gestión del Riesgo de Desastres - UNGRD para la prevención del riesgo en actividades mineras | **A2_2_3** | 35,00 | 56,52 | 62,5 | Baja | Alta | III:Menos  Urgente | 51,34 | 5 |
| Establecer mecanismos de coordinación y colaboración efectivos para el intercambio de información, propuesta de acciones preventivas y el control de trazabilidad, así como para la detección de explotaciones no autorizadas e ilícitas de minerales | **A2_2_4** | 41,79 | 78,70 | 82,5 | Baja | Alta | III:Menos  Urgente | 67,66 | 1 |
| Articular acciones para fortalecer la profesionalización, generación de competencias laborales y capacitación en materia de seguridad minera en el recurso humano vinculado a la actividad minera | **A2_2_5** | 18,57 | 36,09 | 80,0 | Baja | Baja | IV:puede  esperar | 44,89 | 6 |
| Fortalecer el Sistema de Información Minero Colombiano (SIMCO) a través de la actualización de la información del sector minero, como insumo para la unidad de análisis, que permita la toma de decisiones y la adopción de políticas | **A2_2_6** | 42,14 | 74,35 | 82,5 | Baja | Alta | III:Menos  Urgente | 66,33 | 2 |

- Objective 2 Strategic Line 3

| **Acción** | **ID Acción OBJ_LINEA_ACCION** | **Importancia** | **Gobernabilidad** | **Robustez** | **Categoria importancia** | **Categoria Gobernabilidad** | **Categoria Acción** | **AHP total** | **Prioridad** |
| --- | --- | --- | --- | --- | --- | --- | --- | --- | --- |
| Instalar mesas de trabajo interinstitucionales en los distritos mineros delimitados por el Ministerio de Minas y Energía (MME) para el análisis de los componentes sociales, ambientales, mineros, territoriales y demás | **A2_3_1** | 53,57 | 76,67 | 81,3 | Alta | Alta | I:Urgente | 70,50 | 1 |
| Implementar estrategias de comunicación efectivas que faciliten el conocimiento del sector y permitan que la información relevante sobre la actividad minera y los procesos regulatorios sean accesibles al público y a las partes interesadas | **A2_3_2** | 10,00 | 80,00 | 66,3 | Baja | Alta | III:Menos  Urgente | 52,08 | 3 |
| Elaborar periódicamente boletines sectoriales con información relevante de la actividad minera durante el periodo, para el conocimiento integral del sector minero colombiano. | **A2_3_3** | 11,43 | 70,00 | 100,0 | Baja | Alta | III:Menos  Urgente | 60,48 | 2 |
| Mejorar y posicionar la imagen institucional del sector mediante estrategias de comunicación | **A2_3_4** | 30,00 | 40,00 | 81,3 | Baja | Baja | IV:puede  esperar | 50,42 | 5 |
| Crear espacios de capacitación y asesoría en mecanismos de participación ciudadana, en actualizaciones normativas, requisitos, procedimientos y las funciones de las entidades frente al tema minero | **A2_3_5** | 28,93 | 45,00 | 81,3 | Baja | Baja | IV:puede  esperar | 51,73 | 4 |

- Objective 3 Strategic Line 1

| **Acción** | **ID Acción OBJ_LINEA_ACCION** | **Importancia** | **Gobernabilidad** | **Robustez** | **Categoria importancia** | **Categoria Gobernabilidad** | **Categoria Acción** | **AHP total** | **Prioridad** |
| --- | --- | --- | --- | --- | --- | --- | --- | --- | --- |
| Realizar análisis de estrategias de asociatividad, formalización y otras alternativas para promover y fomentar la minería autorizada en el país | **A3_1_1** | 67,50 | 81,82 | 40,0 | Alta | Alta | I:Urgente | 63,11 | 3 |
| Avanzar en el apoyo a procesos de formalización colectiva desde el componente técnico y jurídico | **A3_1_2** | 63,13 | 74,55 | 40,0 | Alta | Alta | I:Urgente | 59,22 | 5 |
| Brindar asistencia técnica a las Unidades Productivas Mineras (UPM) beneficiarias en función de la vocación y tránsito hacia la formalización | **A3_1_3** | 43,75 | 47,27 | 45,0 | Baja | Baja | IV:puede  esperar | 45,34 | 8 |
| Realizar evaluación y seguimiento al Plan Único de Legalización y Formalización Minera | **A3_1_4** | 38,44 | 52,73 | 100,0 | Baja | Alta | III:Menos  Urgente | 63,72 | 2 |
| Diseñar e implementar una estrategia para fortalecer conocimientos y facilitar la comprensión en los trámites para la formalización y/o titulación de la actividad minera en los territorios | **A3_1_5** | 31,25 | 69,09 | 60,0 | Baja | Alta | III:Menos  Urgente | 53,45 | 7 |
| Asistir cuando se requiera a los mineros informales, tradicionales y de pequeña escala en tránsito a la formalización o con alguna figura de formalización en los trámites de creación de esquemas asociativos. | **A3_1_6** | 25,63 | 70,00 | 100,0 | Baja | Alta | III:Menos  Urgente | 65,21 | 1 |
| Implementar estrategias de comunicación y capacitación para fortalecer conocimientos y facilitar la comprensión del trámite administrativo de las Áreas de Reserva Especial, como mecanismo de formalización. | **A3_1_7** | 51,25 | 69,09 | 60,0 | Alta | Alta | I:Urgente | 60,11 | 4 |
| Implementar estrategias de comunicación, capacitación y relacionamiento en y/o con el territorio para fortalecer conocimientos y facilitar la comprensión en los trámites para la formalización y/o titulación de la actividad minera en los territorios. | **A3_1_8** | 18,50 | 70,00 | 80,0 | Baja | Alta | III:Menos  Urgente | 56,17 | 6 |

- Objective 3 Strategic Line 2

| **Acción** | **ID Acción OBJ_LINEA_ACCION** | **Importancia** | **Gobernabilidad** | **Robustez** | **Categoria importancia** | **Categoria Gobernabilidad** | **Categoria**  **Acción** | **AHP total** | **Prioridad** |
| --- | --- | --- | --- | --- | --- | --- | --- | --- | --- |
| Diseñar e implementar un mecanismo de reporte de información periódica que permita suministrar información estructurada sobre las no conformidades en materia de seguridad minera evidenciadas en campo | **A3_2_1** | 21,88 | 80,45 | 100,0 | Baja | Alta | III:Menos  Urgente | 51,16 | 5 |
| Diseño e implementación de una herramienta que permita hacer seguimiento al cumplimiento de las medidas de seguridad minera y de la implementación de los planes de mejoramiento para su levantamiento o cierre. | **A3_2_2** | 36,56 | 76,82 | 100,0 | Baja | Alta | III:Menos  Urgente | 56,69 | 3 |
| Capacitar en estándares de prevención, seguridad minera y metodologías de investigación de accidentes; y entrenamiento de personal para atención de emergencias mineras | **A3_2_3** | 38,44 | 76,82 | 100,0 | Baja | Alta | III:Menos  Urgente | 57,63 | 2 |
| Diseñar guías, protocolos o herramientas prácticas para que se puedan identificar y manejar los riesgos inherentes a la actividad minera; y socializarlas a los trabajadores | **A3_2_4** | 36,56 | 75,00 | 100,0 | Baja | Alta | III:Menos  Urgente | 55,78 | 4 |
| Fortalecer las estrategias de capacitación en preparación y atención de emergencias mineras en el marco de la Gestión de Riesgos de Desastres | **A3_2_5** | 42,19 | 75,00 | 100,0 | Baja | Alta | III:Menos  Urgente | 58,59 | 1 |
| Realizar la adecuación, mantenimiento y mejora continua de las sedes de salvamento minero | **A3_2_6** | 23,13 | 76,82 | 100,0 | Baja | Alta | III:Menos  Urgente | 49,97 | 6 |
| Realizar la adquisición y mantenimiento de equipos especializados para salvamento minero | **A3_2_7** | 23,13 | 75,00 | 100,0 | Baja | Alta | III:Menos  Urgente | 49,06 | 7 |

- Objective 3 Strategic Line 3

| **Acción** | **ID Acción OBJ_LINEA_ACCION** | **Importancia** | **Gobernabilidad** | **Robustez** | **Categoria importancia** | **Categoria Gobernabilidad** | **Categoria Acción** | **AHP total** | **Prioridad** |
| --- | --- | --- | --- | --- | --- | --- | --- | --- | --- |
| Creación de Centros de desarrollo tecnológico y parques - científicos, tecnológicos y de innovación para mejorar las condiciones de productividad y competitividad, optimizar el uso sostenible de los factores productivos, facilitar los procesos de transformación y comercialización (Art. 10 - Ley 2250 de 2022) (Centros de Desarrollo Minero) | **A3_3_1** | 65,63 | 78,18 | 83,8 | Alta | Alta | I:Urgente | 75,85 | 2 |
| Brindar asistencia técnica integral a los mineros tradicionales y de pequeña escala para la consolidación de proyectos mineros viables y sostenibles. | **A3_3_2** | 80,63 | 84,55 | 58,8 | Alta | Alta | I:Urgente | 74,64 | 3 |
| Fomentar la aplicación de buenas prácticas geológico-mineras, sociales y ambientales en el desarrollo de las actividades mineras de pequeña escala, de conformidad con los instrumentos técnicos y normativos desarrollados para la pequeña mineria. | **A3_3_3** | 60,00 | 66,36 | 43,8 | Alta | Alta | I:Urgente | 56,70 | 8 |
| Elaborar las guías metodológicas para el aprovechamiento de minerales estratégicos y críticos sin el uso de sustancias contaminantes (Documentos de lineamientos técnicos sobre beneficio de minerales socio-ambientalmente manejable) | **A3_3_4** | 57,81 | 66,36 | 62,5 | Alta | Alta | I:Urgente | 62,23 | 6 |
| Promover el fortalecimiento en el desempeño de las operaciones mineras de pequeña escala bajo una visión integral con el ambiente y con el territorio. | **A3_3_5** | 57,81 | 62,27 | 78,8 | Alta | Alta | I:Urgente | 66,28 | 5 |
| Socializar las guías minero ambientales de exploración, explotación, transformación y beneficio, así como de formalización y pequeña minería actualizadas, con el fin de promover buenas prácticas minero-ambientales en todas las escalas de la minería a nivel territorial. | **A3_3_6** | 78,13 | 67,27 | 100,0 | Alta | Alta | I:Urgente | 81,80 | 1 |
| Realizar la evaluación y seguimiento del Plan de Acción Nacional sobre mercurio en la minería artesanal y de pequeña escala en Colombia | **A3_3_7** | 51,25 | 62,73 | 58,8 | Alta | Alta | I:Urgente | 57,58 | 7 |
| Implementar una estrategia de seguimiento a los procesos de "debida diligencia en la cadena de suministro" a titulares mineros | **A3_3_8** | 51,25 | 72,27 | 100,0 | Alta | Alta | I:Urgente | 74,51 | 4 |

- Objective 3 Strategic Line 4

| **Acción** | **ID Acción OBJ_LINEA_ACCION** | **Importancia** | **Gobernabilidad** | **Robustez** | **Categoria importancia** | **Categori Gobernabilidad** | **Categoria**  **Acción** | **AHP total** | **Prioridad** |
| --- | --- | --- | --- | --- | --- | --- | --- | --- | --- |
| Crear y fortalecer una herramienta de huella digital de minerales y/o trazabilidad de minerales para determinación de procedencia y uso de sustancias ilícitas en el procesos de aprovechamiento o beneficio. | **A3_4_1** | 56,25 | 64,55 | 58,8 | Alta | Alta | I:Urgente | 59,85 | 4 |
| Fortalecer y consolidar la competencia técnica y red de laboratorios de caracterización de materiales de interés geológico para la generación de conocimiento geocientífico y nuclear del país. | **A3_4_2** | 20,63 | 45,91 | 78,8 | Baja | Baja | IV:puede  esperar | 48,43 | 6 |
| Implementar estrategias para la adopción de prácticas mineras de tecnologías limpias y de última generación mediante transferencia tecnológica en los territorios para avanzar en la reducción de la huella de carbono y mejorar la competitividad | **A3_4_3** | 43,75 | 68,18 | 43,8 | Baja | Alta | III:Menos  Urgente | 51,89 | 5 |
| Incentivar la investigación y desarrollo para la implementación de nuevas tecnologías en la minería, incluyendo economía circular, reciclaje de minerales y uso eficiente de recursos | **A3_4_4** | 71,25 | 75,45 | 78,8 | Alta | Alta | I:Urgente | 75,15 | 2 |
| Estructurar e implementar planes de trabajo para impulsar la eficiencia energética y la autogeneración eléctrica en las áreas mineras tituladas enmarcadas en la hoja de ruta de la transición energética justa | **A3_4_5** | 50,63 | 75,45 | 78,8 | Alta | Alta | I:Urgente | 68,28 | 3 |
| Implementar estrategias que promuevan la adopción de estándares de calidad para dar mayor valor agregado a lo largo de la cadena de producción de minerales | **A3_4_6** | 100,00 | 100,00 | 58,8 | Alta | Alta | I:Urgente | 86,25 | 1 |

- Objective 4 Strategic Line 1

| **Acción** | **ID Acción OBJ_LINEA_ACCION** | **Importancia** | **Gobernabilidad** | **Robustez** | **Categoria importancia** | **Categoria Gobernabilidad** | **Categoria**  **Acción** | **AHP total** | **Prioridad** |
| --- | --- | --- | --- | --- | --- | --- | --- | --- | --- |
| Develop coordination spaces that promote the strengthening and development of value chains that add value to strategic minerals for reindustrialization, energy transition, agricultural development, and public infrastructure. | **A4_1_1** | 100,00 | 100,00 | 81,3 | Alta | Alta | I:Urgente | 93,75 | 1 |
| Elaborar una estrategia para promover el uso de FNCER y de alternativas de energéticos de menores emisiones de gases de efecto invernadero (GEI) aplicadas al sector minero. | **A4_1_2** | 48,08 | 38,00 | 81,3 | Baja | Baja | IV:puede  esperar | 55,78 | 4 |
| Generar información georreferenciada de pequeños mineros, mineros tradicionales y mineros de subsistencia (artesanal) como insumo para la toma de decisiones técnicas y normativas en el marco de la reconversión productiva y/o laboral | **A4_1_3** | 44,23 | 38,00 | 100,0 | Baja | Baja | IV:puede esperar | 60,74 | 2 |
| Formular e implementar una estrategia a nivel territorial para facilitar el acceso a capacitación que permitan el fortalecimiento de habilidades y competencias enfocada al sector minero de pequeña escala y sus cadenas de valor relacionadas | **A4_1_4** | 53,08 | 45,00 | 75,0 | Alta | Baja | II:Retadora | 57,69 | 3 |

- Objective 4 Strategic Line 2

| **Acción** | **ID Acción OBJ_LINEA_ACCION** | **Importancia** | **Gobernabilidad** | **Robustez** | **Categoria importancia** | **Categoria Gobernabilidad** | **Categoria**  **Acción** | **AHP total** | **Prioridad** |
| --- | --- | --- | --- | --- | --- | --- | --- | --- | --- |
| Diseñar e implementar estrategias para atraer, retener, identificar y facilitar la recepción de IED sostenible y con transferencia de tecnología para el desarrollo de encadenamientos productivos y la reindustrialización del país con base en los minerales estratégicos | **A4_2_1** | 39,23 | 50,00 | 100,0 | Baja | Alta | III:Menos  Urgente | 44,62 | 1 |
| Desarrollar acciones para robustecer la oferta de recursos financieros dirigidos a la reconversión productiva y laboral de mineros de subsistencia (artesanal), de pequeña escala y tradicionales | **A4_2_2** | 33,08 | 33,00 | 100,0 | Baja | Baja | IV:puede  esperar | 33,04 | 2 |
| Formular e implementar un programa para el fortalecimiento y la identificación de las fuentes de financiación en actores socio empresariales, población subatendida y no atendida | **A4_2_3** | 32,31 | 30,00 | 100,0 | Baja | Baja | IV:puede  esperar | 31,15 | 3 |

- Objective 4 Strategic Line 3

| **Acción** | **ID Acción OBJ_LINEA_ACCION** | **Importancia** | **Gobernabilidad** | **Robustez** | **Categoria importancia** | **Categoria Gobernabilidad** | **Categoria**  **Acción** | **AHP total** | **Prioridad** |
| --- | --- | --- | --- | --- | --- | --- | --- | --- | --- |
| Desarrollar espacios de articulación que promuevan el fortalecimiento y desarrollo de las cadenas productivas que agreguen valor a minerales estratégicos para la reindustrialización, transición energética, desarrollo agrícola e infraestructura pública. | **A4_3_1** | 33,85 | 38,00 | 81,3 | Baja | Baja | IV:puede  esperar | 51,03 | 5 |
| Implementar una estrategia de aglomeraciones o clúster productivas para la creación de encadenamientos en la actividad minera entre sectores y regiones | **A4_3_2** | 29,23 | 39,00 | 56,3 | Baja | Baja | IV:puede  esperar | 41,49 | 8 |
| Documento que integre las estrategias para el desarrollo de la industria de valor agregado en los territorios, con base en los minerales estratégicos | **A4_3_3** | 9,23 | 20,00 | 100,0 | Baja | Baja | IV:puede  esperar | 43,08 | 7 |
| Desarrollar estrategias para fortalecer las cadenas productivas que agreguen valor a minerales estratégicos para la reindustrialización, transición energética, soberanía alimentaria e infraestructura pública. | **A4_3_4** | 40,77 | 51,00 | 31,3 | Baja | Alta | III:Menos  Urgente | 41,01 | 9 |
| Implementar encadenamientos productivos en mineros-mineras de subsistencia (artesanal), de pequeña escala y tradicionales | **A4_3_5** | 32,31 | 48,00 | 31,3 | Baja | Baja | IV:puede  esperar | 37,19 | 10 |
| Identificar y fortalecer aglomeraciones y cadenas de proveeduría mineras con una visión enfocada en la reindustrialización | **A4_3_6** | 25,38 | 26,00 | 31,3 | Baja | Baja | IV:puede  esperar | 27,54 | 11 |
| Formular e implementar proyectos de eficiencia energética, autogeneración, diversificación productiva y/o reindustrialización en territorios con vocación minera | **A4_3_7** | 40,77 | 35,00 | 56,3 | Baja | Baja | IV:puede  esperar | 44,01 | 6 |
| Construir una hoja de ruta para la promoción de proyectos productivos sostenibles en empresas que hacen parte de los encadenamientos mineros que vinculen la economía circular, la bioeconomía, la eficiencia en el uso de recursos o la gestión del cambio climático, entre otros. | **A4_3_8** | 40,77 | 50,00 | 81,3 | Baja | Alta | III:Menos  Urgente | 57,34 | 2 |
| Implementar estrategias para acercar oferta y demanda de un mineral priorizado. | **A4_3_9** | 38,85 | 48,00 | 100,0 | Baja | Baja | IV:puede  esperar | 62,28 | 1 |
| Identificar mediante estudios sectoriales especializados posibles puntos de congestión o ineficiencia en las cadenas de valor de los mercados en los sectores primario y secundario de la economía relacionados con la industria minera | **A4_3_10** | 11,54 | 49,00 | 100,0 | Baja | Baja | IV:puede  esperar | 53,51 | 3 |
| Evaluar los planes subsectoriales de transición energética, seguridad alimentaria e infraestructura y hábitat | **A4_3_11** | 22,31 | 35,00 | 100,0 | Baja | Baja | IV:puede  esperar | 52,44 | 4 |

- Objective 4 Strategic Line 3

| **Acción** | **ID Acción OBJ_LINEA_ACCION** | **Importancia** | **Gobernabilidad** | **Robustez** | **Categoria importancia** | **Categoria Gobernabilidad** | **Categoria**  **Acción** | **AHP total** | **Prioridad** |
| --- | --- | --- | --- | --- | --- | --- | --- | --- | --- |
| Gestionar proyectos de reconversión laboral y de readecuación ambiental y social para mineros de subsistencia (artesanal), de pequeña escala y tradicionales impactados por la terminación de título mineros y de las Áreas de Reserva Especial. | **A4_4_1** | 30,77 | 46,00 | 52,5 | Baja | Baja | IV:puede  esperar | 43,09 | 4 |
| Implementar la Resolución No. 40279 de 2022 referente a los programas de sustitución de actividades mineras y reconversión o reubicación laboral de los pequeños mineros tradicionales ubicados en ecosistemas de páramos delimitados, en las fases de cierre y desmantelamiento de minas. | **A4_4_2** | 39,23 | 55,00 | 75,0 | Baja | Alta | III:Menos  Urgente | 56,41 | 1 |
| Implementar una estrategia para el fortalecimiento de alternativas productivas y laborales del sector minero en los territorios con vocación minera | **A4_4_3** | 32,31 | 60,00 | 52,5 | Baja | Alta | III:Menos  Urgente | 48,27 | 3 |
| Desarrollar programas para ampliar la oferta institucional dirigida a la reconversión productiva y laboral de mineros de subsistencia (artesanal), de pequeña escala y tradicionales | **A4_4_4** | 35,38 | 66,00 | 52,5 | Baja | Alta | III:Menos  Urgente | 51,29 | 2 |
