# Supplementary material for: Strategic Prioritization of Mining Policies in Colombia Through The IGOR Hybrid Framework
Source: Environ Manage. 2026 Jun 11;76(6):213. doi: 10.1007/s00267-026-02500-6 (PMC13260195; doi:10.1007/s00267-026-02500-6)
Supplement: Supplementary file 5 — Supplementary Material_2 [file 267_2026_2500_MOESM5_ESM.docx]

**GUIA RAPIDA PLANTILLA IGOR**

Se cuenta con una plantilla IGOR para cada uno de los objetivos estratégicos.

Se recomienda copiar la plantilla suministrada y cambiarle el nombre a “*Taller IGOR UPME Objetivo X APELLIDO.xlsm”.*

**Hoja Inicio:**

Esta es la guía de la secuencia de pasos.

Tiene vínculos directos a cada una de las otras hojas y de estas también se puede volver a Inicio directamente, cuando complete los diferentes pasos. Ponga clic cuando la complete para mejor seguimiento del cumplimiento de los pasos.

Llene su nombre, entidad u organización a la que representa y la fecha.

**Hoja 1. Priorización de criterios**

Se ofrece una lista de criterios que definirán la Importancia y la Gobernabilidad de cada acción (considerando el objetivo estratégico en cuestión).

Usted solo debe evaluar qué tan importante es cada criterio (respecto al grupo, es decir importancia relativa) para evaluar, más adelante, cada una de las acciones: Se consideran los siguientes niveles de importancia de los **criterios**:

1. Nada importante

2. Apenas importante

3. Medianamente importante

4. Muy importante

5. Absolutamente importante

Estos niveles ya están predefinidos en una lista desplegable. No debe escribir nada diferente a estos.

Si se le hace difícil esta diferenciación, una guía útil es asignar un orden de 1 a n (siendo n el número de criterios), donde 1 representa el criterio más importante del grupo. Con base en esa secuencia, elija los niveles de importancia en la tabla.

**Hoja 2. Priorización de Incertidumbres**

Para cada una de las variables inciertas (o incertidumbres) debe evaluar qué tan impactante sería un valor amenaza sobre el Objetivo estratégico en cuestión y que probabilidad tendrá ese suceso.

Debe elegir de la lista desplegable únicamente.

Niveles de Impacto:

1. Insignificante

2. Menor

3. Moderado

4. Mayor

5. Catastrófico

Niveles de probabilidad:

1. Raro (<20%)

2. Improbable (20-39%)

3. Posible (40-59%)

4. Probable (60-79%)

5. Casi seguro (80-99%)

La prioridad de cada variable incierta se autocalcula. Usted no debe introducir nada en esa columna. Se destaca con colores desde las más catastróficas (rojo) hasta las de prioridad Baja (verde). Se adiciona una gráfica de calor donde las incertidumbres con mas alto impacto y probabilidad están en la zona roja (catastróficas), etc.

**Hola 3. Análisis de robustez**

En esta hoja solo se evalúan impactos sobre el logro del objetivo en cuestión, de las variables inciertas consideradas catastróficas en el paso anterior.

Para cada una de estas se ingresan dos valores:

- Si se implementara la acción i, evalúe entre 0 y 100% qué tanto se logra el objetivo estratégico si se presenta la Peor eventualidad posible de esa incertidumbre?
- Si se implementara la acción i, evalúe entre 0 y 100% qué tanto se logra el objetivo estratégico si se presenta la mejor eventualidad posible de esa incertidumbre?

Los análisis de sensibilidad y robustez se autocalculan, así que usted no debe incluir ninguna información adicional.

El tornado muestra los logros de la acción en presencia de los diferentes escenarios de cada variable incierta. la longitud de las barras representa la sensibilidad. La longitud de cada barra se refiere a que tan sensible son las acciones de la línea estratégica ante los diferentes

Eventos de esa variable. Entre más corta menos sensible. La robustez tiene que ver con que parte de esa barra esta en la zona de satisfacción (cumple como mínimo una meta de logro, por defecto, la meta es 50%).

**Hoja 4.k Consecuencias Objetivo O, Línea estratégica k**

Se debe llenar una hoja por cada línea estratégica.

Cada hoja tiene dos matrices, una con los criterios de Importancia, y otra con los de Gobernabilidad.

Usted debe llenar, con valores de 0 a 100%, que tanto satisface la implementación de la **acción** de la fila al criterio de la columna. Todos los criterios se definieron tal que entre mayor valor mejor el resultado.
